# Draft genome sequence of adzuki bean, *Vigna angularis*

**DOI:** 10.1038/srep08069

**Published:** 2015-01-28

**Authors:** Yang Jae Kang, Dani Satyawan, Sangrea Shim, Taeyoung Lee, Jayern Lee, Won Joo Hwang, Sue K. Kim, Puji Lestari, Kularb Laosatit, Kil Hyun Kim, Tae Joung Ha, Annapurna Chitikineni, Moon Young Kim, Jong-Min Ko, Jae-Gyun Gwag, Jung-Kyung Moon, Yeong-Ho Lee, Beom-Seok Park, Rajeev K. Varshney, Suk-Ha Lee

**Affiliations:** 1 Department of Plant Science and Research Institute for Agriculture and Life Sciences, Seoul National University, Seoul 151-921, Korea; 2 Indonesian Center for Agricultural Biotechnology and Genetic Resources Research and Development (ICABIOGRAD-IAARD), Jalan Tentara Pelajar No. 3A Bogor 16111, Indonesia; 3 Program in Plant Breeding, Faculty of Agriculture at Kamphaeng Saen, Kasetsart University, Kamphaeng Saen, Nakhon Pathom 73140, Thailand; 4 National Institute of Crop Science, Rural Development Administration, Suwon, 441-857, Korea; 5 Research Policy Bureau, R&D Performance Evaluation & Management Division, Nongsaengmyeong-ro 300, Wansan-gu, Junju, 560-500, Korea; 6 International Crops Research Institute for the Semi-Arid Tropics, Patancheru, Andhra Pradesh, India; 7 Soybean Research Team, Legume & Oil Crop Research Division, Jeompiljae-ro 20, Miryang, Gyeongnamdo, 627-803, Korea; 8 National Agrobiodiversity Center of NAAS, RDA, SuwonxGyeongnamdo, Korea; 9 Agricultural Genome Center, National Academy of Agricultural Science, Rural Development Administration, Suwon, 441-707, Korea; 10 Plant Genomics and Breeding Institute, Seoul National University, Seoul, 151-921, Korea

## Abstract

Adzuki bean (*Vigna angularis* var. *angularis*) is a dietary legume crop in East Asia. The presumed progenitor (*Vigna angularis* var. *nipponensis*) is widely found in East Asia, suggesting speciation and domestication in these temperate climate regions. Here, we report a draft genome sequence of adzuki bean. The genome assembly covers 75% of the estimated genome and was mapped to 11 pseudo-chromosomes. Gene prediction revealed 26,857 high confidence protein-coding genes evidenced by RNAseq of different tissues. Comparative gene expression analysis with *V. radiata* showed that the tissue specificity of orthologous genes was highly conserved. Additional re-sequencing of wild adzuki bean, *V. angularis* var. *nipponensis*, and *V. nepalensis*, was performed to analyze the variations between cultivated and wild adzuki bean. The determined divergence time of adzuki bean and the wild species predated archaeology-based domestication time. The present genome assembly will accelerate the genomics-assisted breeding of adzuki bean.

Adzuki bean (*Vigna angularis* var. *angularis*) is a diploid legume crop (2n = 2x = 22) with an estimated genome size of 538 mega bases (Mb)^1^. It is one of the Asian *Vigna* in the Ceratotropis subgenus, under the papilionoid subfamily of the Fabaceae^2^. Adzuki bean is widely cultivated in East Asian countries like China, Japan, and Korea as an ingredient for traditional dessert cuisines due to its sweet taste, as well as its nutritious protein and starch contents. The annual cultivation area for adzuki bean in China, Japan, Korean peninsula, and Taiwan is estimated to be 670,000, 120,000, 30,000, and 20,000 ha, respectively^3^. The wild species of adzuki bean such as *V. angularis* var. *nipponensis*, *V. nakashimae*, and *V. nepalensis*, are widely distributed across East Asia and Himalayan countries^2^. However, archaeological evidences suggested multiple domestication origins in northeast Asia^4^.

Several important legume crops and model plants have been sequenced. This includes warm-season legumes such as *Glycine max*, *Phaseolus vulgaris*, *Cajanus cajan*^5^,^6^,^7^, and *Vigna radiata*^8^. *V. angularis* var. *angularis* is a close relative of *V. radiata* and is adapted to sub-tropical and temperate climate zone. In spite of its economic importance and the demands for improved *V. angularis* var. *angularis* variety, genomic studies for agriculturally important traits and efficient breeding methods for this species have been lacking. Elucidation of the genome sequence of *V. angularis* var. *angularis* could reveal the general genome structure and evolution of this legume species in comparison to closely related genomes and greatly assist comparative genomics of *V. angularis* var. *angularis* and other well-studied legume genomes. In addition, the re-sequencing efforts of cultivated and wild adzuki beans will facilitate the measurement of genetic diversity of each locus and the development of useful markers for putative domestication-related loci.

Here, we assembled a draft genome of adzuki bean into pseudo-chromosomes using sequence data from next generation sequencing. This adzuki bean genome was compared to other warm-season legumes to study genome evolution and speciation. Well established quantitative trait loci (QTLs) of *G. max* were translated into the adzuki bean genome using gene order conservation. In addition, we sequenced two wild adzuki bean species, *Vigna angularis* var. *nipponensis* and *Vigna* *nepalensis*, and identified the putative loci related to domestication in order to develop SNP markers for more efficient breeding. Our genome sequence of *V. angularis* var. *angularis* and QTL-associated genetic markers will boost genomics of warm season legumes and breeding programs of adzuki bean.

## Results

### Genome assembly

*V. angularis* var. *angularis* has a diploid genome. Based on flow cytometry analysis the genome size was estimated to be 612 Mb (Supplementary Table S1) which is higher than the previous estimate of 538 Mb^1^. The selected domesticated line for sequencing was Gyeongwon, a widely grown variety in Korea, which was developed by the Rural Development Administration (RDA) in Korea to reduce root lodging and to improve grain quality.

For *de novo* genome assembly, we prepared two paired-end libraries with 180 bp insert size, along with two 5 kb mate pair, and one 10 kb mate-pair libraries for 100 bp short read sequencing using the Illumina HiSeq 2000 (Supplementary Table S2). A single linear library was also constructed for sequencing using the Roche GS-FLX+ producing total 1,288,628 reads with average read length, 458 bp. Approximately 291-fold sequence read coverage of the estimated genome size was generated by the two sequencing platforms. For reads generated by the Illumina HiSeq 2000, ALLPATHS-LG assembler was used for *de novo* assembly^9^. The long reads generated by GS FLX+ were assembled using Newbler software and the resulting contigs were transformed into paired end reads with 180 bp insert and used as input for ALLPATHS-LG assembly. Using Jellyfish^10^ software at 22 k-mer frequency, we estimated the genome size to be 591 Mb which is close to the size calculated using flow cytometry analysis (Supplementary Fig. S1). The assembly produced 3,883 scaffolds with proper read coverage statistics of sequencing libraries including the pseudo-library from Newbler assembly and the N50 length of the scaffolds was 703 kb (Supplementary Table S3). The sum of the scaffold length was approximately 443 Mb covering 75 percent of the estimated genome size (Supplementary Table S4).

To link the scaffolds into super-scaffolds, we utilized the synteny relationship between *V. angularis* and closely related legume genomes such as *Phaseolus vulgaris*^7^ and *V. radiata* var. *radiata*. This was based on the assumption that the gene order among closely-related warm season legume species is highly conserved. Identification of the synteny relationship and calculation of the Ks values for each orthologous or paralogous gene pair was performed using MCSCANX software^11^. We retrieved the synteny blocks from the most recent peak in the Ks frequency plot (Supplementary Fig. S2) and extracted conserved genomic blocks that showed multi-species collinearity among the three legume genomes to be used as bridges for super-scaffolding (Supplementary Fig. S3). The synteny-based scaffolding strategy improved the N50 length from 704 kb to 1.5 Mb and the maximum length of scaffolds from 4.4 Mb to 11.1 Mb (Supplementary Table S5).

To assemble the pseudo-molecules, we implemented the genotyping by sequencing (GBS) method^12^ to construct a high-density genetic map of adzuki bean. The mapping population comprised of 133 F_4_ lines generated by single-seed descent from a cross between *V. angularis* var. *angularis* (Gyeongwon) and the wild species *V. nakashimae* (IT178530). GBS short reads were mapped to genomic regions flanked by ApeKI restriction sites using Bowtie2 software^13^. A total of 4,524 segregating SNP sites were identified. Possible co-segregating sites within 1 kb regions were merged reducing the total SNP number to 2,347. However, more than half of the SNPs showed segregation distortions that are probably due to interspecific crossing (Supplementary Table S6). Removal of the distorted SNPs resulted in a high-density genetic map with 814 SNPs in 11 linkage groups (Supplementary Fig. S4 and Supplementary Table S7). In total, 158 scaffolds could be anchored to the genetic map to construct the 11 pseudo-chromosomes. The sum of the anchored scaffolds was 210 Mb and the length of N50 was 25 Mb (Supplementary Table S8). However, 78 scaffolds and super-scaffolds were anchored without orientation information, because only a single marker could be used to anchor them to the map (Supplementary Table S4). Total 45 super-scaffolds were anchored to genetic map and 43 super-scaffolds were consistent with our genetic map suggesting the reliability of the synteny-based scaffolding method (Supplementary Fig. S3 and Supplementary Table S9).

### Prediction of genes and repetitive sequences in adzuki bean

**Figure 1:**
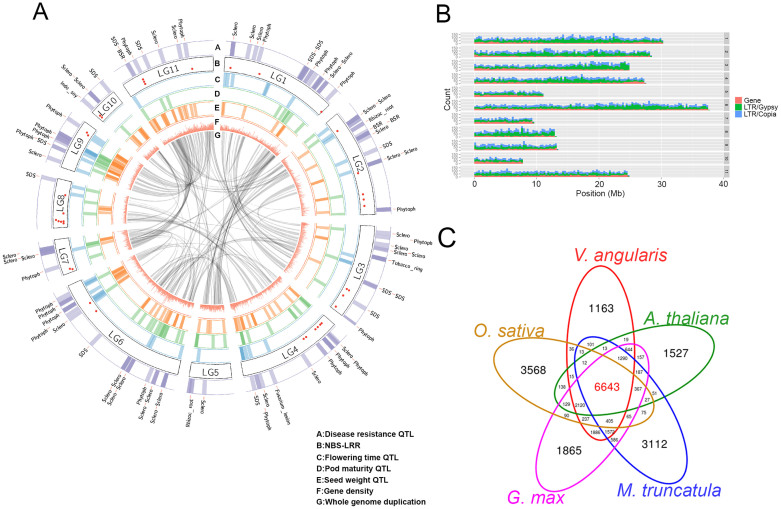
Summary of genome assembly of cultivated adzuki bean and analyses of genes, repetitive sequences, and predicted QTLs. (A) Circular map showing predicted QTL regions of *V. angularis* var. *angularis* based on the synteny relationship with *G. max* and the WGD regions displayed by the grey ribbons at most inner part of circle. From outer to inner layer, four types of QTLs are depicted (disease resistance, flowering time, pod maturity and seed weight, respectively). Highlighted region of each layers represent the candidate physical position of the related QTLs. The red dots on the chromosomes represent the loci of NBS-LRR domain containing genes. (B) The distribution of the predicted genes and the repetitive sequences in adzuki bean genome plotted by 300 kb bin length. The colored bars represent the counts of genes and repeated elements in each bin; LTR/Gypsy (green), LTR/Copia (blue), LINE (blue), and genes (red). (C) Venn diagram depicting the clustering analysis of the five protein sets from *A. thaliana*, *M. truncatula*, *G. max*, *O. sativa*, and *V. angularis*. The numbers of homolog clusters were indicated for each species and species intersection.

After masking the identified repetitive sequences, we implemented the structural and homology-based gene prediction procedure according to the MAKER pipeline^14^,^15^. In order to obtain direct evidence of gene expression, we extracted mRNA from adzuki bean flower, pod, leaf, and root tissues (Supplementary Table S10). The mRNA samples were sequenced using the Illumina HiSeq 2000 for subsequent *de novo* assembly using Trinity software^16^. The assembled contigs were pooled and supplied to the MAKER pipeline as the evidence of transcription and 26,857 high-confident genes were predicted. Using CEGMA pipeline, more than 86 percent of 248 core eukaryotic genes (CEG) could be completely matched to our genome assembly, and the 99 percent of 248 CEGs matched to the predicted proteins using BLASTP algorithm with *E*-value 1e-10 (Supplementary Table S4)^17^. To test the reliability of the predicted *V. angularis* gene set, we compared the sequence length distributions of the genes, coding DNA sequences (CDS), and introns to the gene models of *P. vulgaris*, *G. max*, and *V. radiata.* The density plot showed consistent distributions of CDS and intron length among the three legume genomes. However, the proportion of short genes (~250 bp) was higher in *V. angularis* (Supplementary Fig. S5). Of the 26,857 high-confident genes, 15,976 were located on pseudo chromosomes (Fig. 1a, 1b and Supplementary Table S4). Clustering analysis was performed on the protein sets of *V. angularis* var. *angularis* and the protein sequences of *A. thaliana*, *M. truncatula*, *O. sativa,* and *G. max* using OrthoMCL software^18^, and identified 6,643 gene clusters that are shared among all five species and 1,163 clusters that are specific to *V. angularis* var. *angularis* (Fig. 1c, Supplementary Table S11). We could assign functional annotations to 21,532 genes using InterProScan and BLAST against Arabidopsis proteins (Supplementary Table S4).

**Figure 2:**
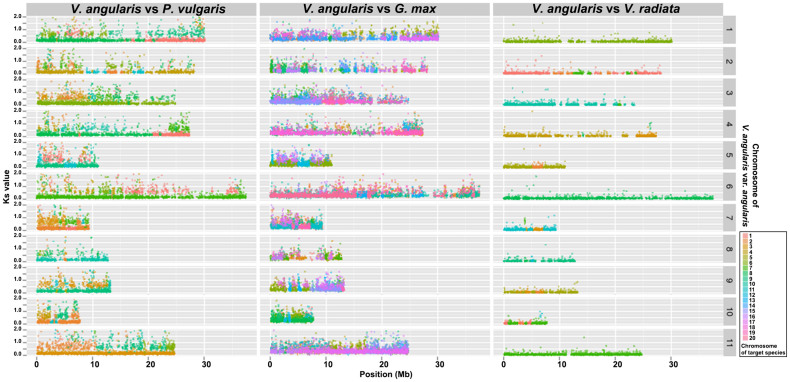
Visualization of the synteny relationships among the closely related warm season legumes, *V. angularis*, *V. radiata*, *P. vulgaris* and *G. max*. The x-axis indicates chromosomal locations of genes in synteny relationship, and the y-axis indicates Ks value of the corresponding gene pair, showing both conservation of gene order as well as chromosomal rearrangements of synteny blocks.

The predicted gene content of *V. angularis* var. *angularis* showed extensive synteny relationship with closely-related warm season legumes including *G. max*, *P. vulgaris*, and *V. radiata* (Fig. 2 and Supplementary Fig. S6). We examined the tissue specificity of gene expressions in *V. angularis* var. *angularis* and *V. radiata* var. *radiata* using RNA-Seq data from four different tissues (Supplementary Table S10 and Supplementary Fig. S7). There are 9,196 orthologs between *V. angularis* var. *angularis* and *V. radiata* var. *radiata* that showed persistent tissue specificity, suggesting that gene functions were extensively retained even after speciation (Supplementary Table S12).

Using the Pfam annotations of each protein, we classified transcription factors according to the rules described in Lang et al.^19^. In total, 2,669 genes encoding transcription factors (TFs) were identified in the adzuki bean genome. We compared the relative TF abundance with that of other plant genomes (Supplementary Table S13) and found the overall proportions of TF gene families to be similar in these plant genomes. However, bZIP2 consistently makes up less than 1% of the total TFs in legume genomes. This contrasts with non-legume genomes such as *A. thaliana*, *Z. mays*, *O. sativa*, and *B. distachyon* where bZIP2 represented more than 3% of all TFs, suggesting a possible gene loss event of this TF family in the common ancestor of legume plants (Supplementary Table S13).

We surveyed the repetitive sequences within the scaffold sequences to examine the abundance and distribution of transposable elements, which are known as major drivers of genome evolution^20^. Homology- and structure-based analysis revealed approximately 43.1% of sequenced adzuki bean genome as repetitive sequences (Supplementary Table S14). As in the other legumes and in that of the closely related *V. radiata*, the predominant repetitive sequences were annotated as long terminal repeat (LTR) retrotransposons^5^,^6^,^21^,^22^. Among the LTR retrotransposons, Gypsy and Copia constituted 19% and 10% of the adzuki bean genome sequences, respectively (Supplementary Table S14). DNA transposons, CACTA, Mutator, PIF-Harbinger, hAT, Helitron, MULE-MuDR, and Tc1-Mariner, were also detected, comprising approximately 2.7% of the adzuki bean genome sequences.

### Domestication traces of adzuki bean

Human selection activities on crops have inflicted major effects on crop genomes, which ultimately result in domestication syndrome, which is marked by loss of seed shattering, minimization of seed dormancy, and an increase in both seed size and number^23^. To identify the domestication traces within the genome of *V. angularis* var. *angularis*, we sequenced wild relatives of adzuki bean, *V. nepalensis* (AusTRCF85148) and *V. angularis* var. *nipponensis* (IT241912) (Supplementary Table S2). Single paired-end libraries from each genotype were sequenced using Illumina HiSeq 2000 generating about 50 Gb of short reads which represents more than 85-fold coverage of the adzuki bean genome. We also analyzed short read sequences of wild adzuki bean, *Vigna nakashimae* (IT178530), which was generated in our previous study^24^.

The 22-base k-mer frequency analysis of these short read sequences revealed a variation of genome size among wild adzuki beans (Supplementary Fig. S1). This is especially true for *V. nakashimae*, which showed a much larger genome size than *V. angularis* var. *angularis* and the rest of the wild adzuki beans.

We mapped the short read sequences onto our adzuki bean reference genome using Burrows-Wheeler Aligner software (Supplementary Table S15 and S16)^25^. *V. angularis* var. *nipponensis* had 667,097 SNPs compared with the reference genome and the SNP frequency to total mapped regions (SNPs per kb) was 1.82. 75,476 of the identified SNPs are located within coding regions and 3,840 of those SNPs could potentially cause non-synonymous protein sequence changes in 1,421 gene products. We also identified 97, 932 insertions and deletions (INDELs) of which 14,033 were in coding regions.

*V. nepalensis* showed much higher polymorphism than *V. angularis* var. *nipponensis* as demonstrated by the presence of 3,511,378 SNPs (10.24 SNPs per kb). The number of SNPs in coding region was 433,210, and there were 18,034 non-synonymous SNPs that affected 6,464 genes. A total of 410,232 INDELs were identified and 71,337 were within coding regions.

*V. nakashimae* showed 3,342,795 SNPs and 624,856 intragenic SNPs. However, the ratio of aligned reads to total reads and the predicted number of INDELs were notably lower than those of other wild adzuki beans. This could be due to the difficulty of read mapping between the reference species (*V. angularis*) and the highly diverged species (*V. nakashimae*) showing notable genome size discrepancy (Supplementary Fig. S1).

**Figure 3:**
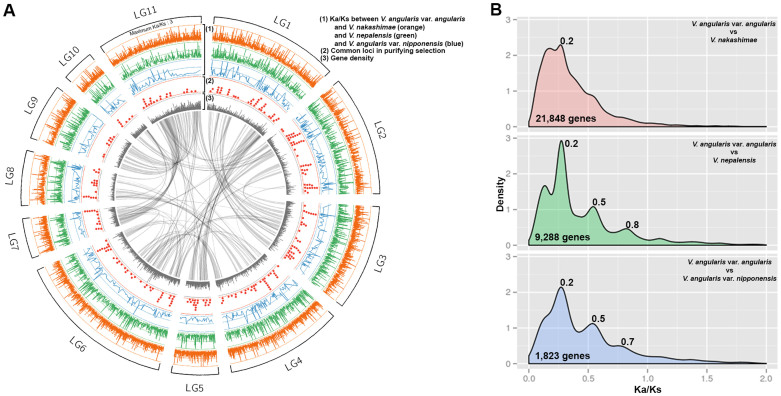
Selection pressure for each locus in adzuki bean genome between cultivated (*V. angularis* var. *angularis*) and wild adzuki beans (*V. angularis* var. *nipponenesis*, *V. nepalensis*, and *V. nakashimae*). (A) Circos map displaying Ka/Ks value of each locus (from outer most layer), the positions of common loci in purifying selection, and gene density. The most inner ribbons indicate the duplicated synteny blocks (B) Density plot showing the distribution of Ka/Ka values between cultivated and wild adzuki beans.

We compared orthologs between cultivated and wild adzuki bean to elucidate domestication-related loci. The orthologous coding sequences of *V. angularis* var. *nipponensis* and *V. nepalensis* were reconstructed by substituting the coding sequences of *V. angularis* var. *angularis* with their respective SNP data. Since *V. nakashimae* short reads were not efficiently mapped onto our reference genome, we performed *de novo* assembly of the short read sequences using ABySS software^26^ and implemented gene prediction (Supplementary Table S4). From the ortholog comparison between cultivated and wild adzuki bean, we calculated the ratio of the number of nonsynonymous substitutions per non-synonymous site (Ka) to the number of synonymous substitutions per synonymous site (Ks) to estimate the selective pressure on each gene (Fig. 3a). *V. angularis* var. *nipponensis* showed the lowest number of polymorphic genes (1,823) in the Ka/Ks calculation. We observed one consistent peak at Ka/Ks value of 0.2 suggesting purifying selection between cultivated and three wild adzuki beans (Ka/Ks < 1)^27^ (Fig. 3b). Notably, the Ka/Ks distributions of *V. angularis* var. *angularis* x *V.angularis* var. *nipponensis* and *V. angularis* var. *angularis* x *V. nepalensis* were highly similar consistently showing three peaks (0.2, 0.5, and 0.7 ~ 0.8). In both comparisons, a total of 307, 152, and 75 genes were commonly found within the three peaks, respectively (Supplementary Table S17). This suggests three different degrees of selection pressure on these loci between cultivated and wild adzuki bean. Even though a Ka/Ks of less than 1 has been interpreted as a signature of purifying selection^27^, a subset of the genes in each peak can be candidates for explaining the difference between wild and cultivated adzuki bean, such as speciation and domestication (Supplementary Table S17). For example, the homologs to disease related genes in the second (0.4 ~ 0.6) and third peak (0.6 ~ 0.9) such as Vang03g15160, Vang02g14420, Vang0291s00070, Vang0229s00140, Vang02g14420 may possess novel disease resistance alleles in wild adzuki beans which are distinct to those in cultivated adzuki bean (Supplementary Table S17).

### Marker development and its utilization in breeding program

In order to produce genetic markers that are easily applicable for QTL mapping and marker assisted breeding programs, we identified simple sequence repeat (SSR) markers using MISA software^28^. A total of 143,113 SSRs were detected and the number of tri-repeat unit SSRs, the preferred type for genotyping, were 1,941 (Supplementary Table S18).

We predicted the associated QTLs to these SSR markers using translational genomics approach^29^. *G. max* is a warm-season model legume crop closely related to *V. angularis* var. *angularis* showing co-linearity of most of the gene content (Fig. 2). Hence, the predicted QTLs from the comparison between *G. max* and *V. angularis* would be useful clue to determine genomic regions related to agriculturally important traits in *V. angularis* var. *angularis* genome^30^. We translated the genomic positions of 2,010 QTL-associated SSR markers of *G. max* to corresponding genomic positions of *V. angularis* by 569 orthologous synteny blocks (Supplementary Table S19) and plotted the agriculturally important QTLs such as flowering time, maturity, seed size, yield, and disease resistance onto a circular map (Fig. 1a). The disease resistance QTLs were likely to be around the 87 genes that code for nucleotide-binding site (NBS) and leucine-rich repeat (LRR) domains, which are commonly associated with disease resistance (Fig. 1a and Supplementary Table S20)^31^. The flanking markers of these translated QTLs can be used for breeding programs (Supplementary Table S19). We also constructed a database containing the gene information, genetic markers, and associated QTL data in Jbrowse environment, which can be accessed at http://plantgenomics.snu.ac.kr/ (Supplementary Fig. S8).

### Genome evolution of adzuki bean

**Figure 4:**
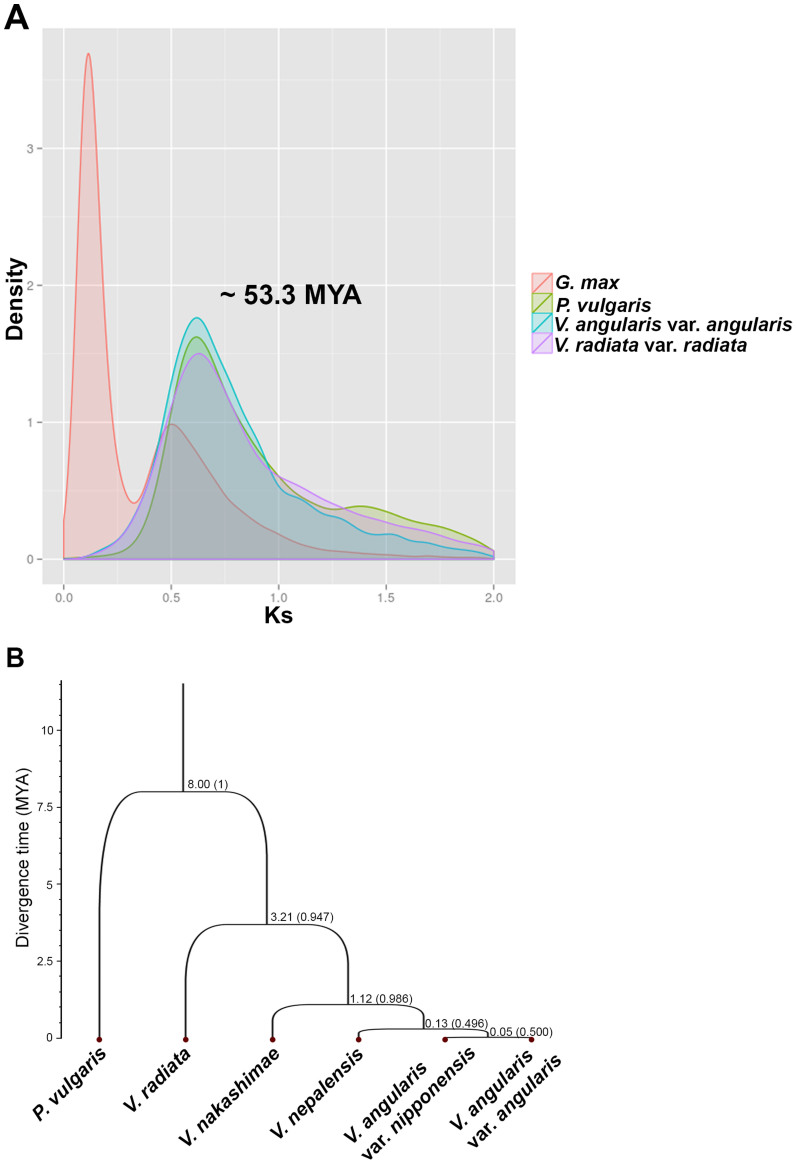
Analyses of the evolution of adzuki bean with comparison to closely related warm season legumes. (A) Estimation of WGD using the density plot of Ks values within each gene set of *V. angularis* (blue), *P. vulgaris* (green), *V. radiata* (purple), and *G. max* (red). (B) Species tree of cultivated adzuki bean (*V. angularis* var. *angularis*), wild adzuki beans (*V. angularis* var. *nipponensis*, *V. nepalensis*, and *V. nakashimae*), *V. radiata*, and *P. vulgaris*. The divergence times of each nodes were estimated by a Bayesian MCMC method calibrated by the root divergence time (8 MYA) between *Phaseolus* and *Vigna*. The posterior probability of each node is depicted in parentheses.

Using 60 orthologs of *P. vulgaris*, *V. radiata*, *V. nakashimae*, *V. nepalensis*, *V. angularis* var. *nipponensis*, and *V. angularis* var. *angularis*, we constructed a species tree (Fig. 4) (Supplementary Table S21). *V. angularis* var. *angularis* formed a distinct clade that included the wild adzuki beans in the species tree. As expected from genome size difference, *V. angularis* var. *nipponensis* was closest to *V. angularis* var. *angularis*, whereas *V. nakashimae* was more diverged. We estimated the speciation times, which were calibrated using the divergence time, 8.0 million years ago (MYA), between *Phaseolus* and *Vigna*^32^. For our sampled accessions, the minimum speciation time between cultivated and wild adzuki bean was 0.05 MYA, which predated the archaeological evidence for adzuki bean cultivation (~5,000 years before present)^4^. The Ks density plot calculated using synteny relationship within the legume genomes revealed the single ancient whole genome duplication at ~53.3 MYA based on the substitution rate, 6.1 × 10^−9^ 33, which is commonly shared among *V. angularis*, *V. radiata*, and *P. vulgaris* (Fig. 4). We identified 1,273 tandemly duplicated genes in the adzuki bean genome; these genes are highly enriched in the gene ontology categories of defense response, oxidation reduction, and phosphorylation, which is consistent with findings in other plant genomes (Supplementary Table S22 and Supplementary Fig. S9)^34^,^35^.

## Discussion

Among Asian *Vigna*, adzuki bean is an economically important grain legume due to its nutritional properties and popular use in dessert foods. A better understanding of adzuki bean genetics is important for more efficient breeding and in light of an increase in biotic and abiotic stresses on crops that may accompany climate change. The adzuki bean reference genome sequence and re-sequencing efforts of two different wild adzuki beans, *V. angularis* var. *angularis* and *V. nepalensis*, presented in this manuscript, are a rich source of genetic markers, loci under degrees of selection pressure, and putative candidate genes for several agriculturally important traits that were derived from translational genomics with *G. max* (Fig 1a, Fig 3a and Supplementary Table S19).

Plant genome complexity and the length of repeats that often exceed the insert size of present mate pair library technology can limit the *de novo* assembly of NGS reads to a certain saturation point. We largely improved the assembly by implementing the synteny-based scaffolding approach using gene order conservation between closely related legume species (*V. angularis* var. *angularis*, *V. radiata* var. *radiata*, and *P. vulgaris*). Thus, this method could be used as an alternative to the longer sequencing reads or mate pair library with larger insert size solving the assembly problem. In addition, we anchored super-scaffolds onto the genetic map constructed using the GBS-based genotypes of F_4_ recombinant inbred lines of *V. angularis* var. *angularis* and *V. nakashimae*. Due to the segregation distortion, relatively small amounts of GBS-derived SNPs were used for constructing the genetic map. The possible cause of this segregation distortion in this population is the genome size difference of parental lines, which may result in ectopic recombination (Supplementary Fig. S1)^36^.

Even in our limited number of cultivated and wild adzuki bean samples, we could catch a glimpse of the speciation time between *V. angularis* var. *angularis* and *V. angularis* var. *nipponensis*, which occurred at around 50,000 years ago. As the domestication time evidenced by archaeology was ~5,000 before present^4^, we could hypothesize that the domestication event by start of human cultivation occurred after speciation between wild and cultivated adzuki bean. Additional population-level re-sequencing efforts of cultivated and wild adzuki beans would reveal the speciation time and also the domestication sites at higher confidence level, and also test the previous hypothesis of multiple domestication sites of *V. angularis*^4^.

## Methods

### Plant materials

The cultivated adzuki bean accession, Gyeongwon (*V. angularis* var. *angularis*, IT213134), and wild adzuki bean accession (*V. angularis* var. *nipponenesis*, IT241912) were provided by the Rural Development Administration (RDA) Genebank Information Center in Korea. The other wild adzuki bean (*V. nepalensis*, AusTRCF85148) was sourced from the Australian Collections of Plant Genetic Resources in Australia.

### Genome assembly and gene prediction

For genome size estimation, we used Jellyfish^10^ software to observe K mer (22-mer for this study) frequency distribution by “jellyfish count” command with parameter -C, -m 22, -s 50G, -t 10, and -c 6. “jellyfish histo” command created the histogram of K mer frequency and the peak frequency could be observed. The sequencing depth (N) was estimated by the equation: N = M * L/(L − K + 1) where M is peak K mer frequency, L is read length, K is K mer length.

We also estimated the genome size by the flowcytometry analysis using procedure modified from Arumuganathan and Earle *et al.*^37^. Briefly, the procedure consists of preparing suspensions of intact nuclei by chopping of 50 mg adzuki bean leaf tissues in MgSO_4_ buffer mixed with DNA standards and stained with propidium iodide in a solution containing DNAase-free-RNAase. Fluorescence intensities of the stained nuclei are measured by FACScalibur flow cytometer (Becton-Dickinson, San Jose, CA). Values for nuclear DNA content are estimated by comparing fluorescence intensities of the nuclei of the testing sample with those of an appropriate internal DNA standard such as nuclei from Chicken Red blood cells (2.5 pg/2C), *Glycine max* (2.45 pg./2C), *Oryza sativa* cv. Nipponbare (0.96 pg/2C), or *Arabidopaia* *thaliana* (0.36 pg/2C). For each measurement, the propidium iodide fluorescence area signals (FL2-A) from 1000 nuclei were collected and analyzed by CellQuest software (Becton-Dickinson, San Jose, CA).

For genome assembly of cultivated adzuki bean, we used the Illumina HiSeq 2000 using types of sequencing libraries such as two fragment libraries and two 5 kb and one 10 kb mate pair libraries. One additional single linear library was sequenced using the Roche GS-FLX+ (Supplementary Table S2 and Supplementary Table S23). The assembles of Illumina HiSeq 2000 and GS-FLX+ read sequences were implemented using ALLPATHS-LG^9^ and Newbler, respectively. ALLPATH-LG was run with default parameter and Newbler was run with option -large and -trim for “runProject” command. The contigs generated by Newbler were chopped into pseudo fragment library reads and supplied to ALLPATHS-LG again. Software ABYSS was used for the assembly of *V. nakahsimae* using the option k = 84 and q = 20 for “abyss-pe” command. The traces of repetitive sequences were searched using LTR-harvest^38^ and TransposonPSI (http://transposonpsi.sourceforge.net/) with default parameters. The captured repetitive sequence blocks were annotated by LTR-digest^39^ using a set of hmm signatures: PF03078.8, PF00385.17, PF01393.12, PF04094.7, PF07253.4, PF00552.14, PF05380.6, PF00077.13, PF08284.4, PF00078.20, PF07727.7, PF06815.6, PF06817.7, PF03732.10, PF00075.17, PF01021.12, PF04195.5, PF00692.12, PF00692.12, and PF00098. The hmm signature of AP_ty1copia and AP_ty3gypsy elements was built using their alignment information from GyDB^40^. We further utilized the *G. max* TE classification based on sequence homology^41^. The predicted and annotated repetitive sequence blocks were supplied to RepeatMasker software as adzuki bean repetitive sequence library^14^.

Adzuki bean gene contents were predicted following MAKER pipeline^15^. RNA-Seq using Illumina HiSeq 2000 were implemented on mRNAs of the four different tissues (leaf, flower, root, and pod) of adzuki bean and *de novo* assembled using Trinity software with default parameter^16^. Contigs were pooled from *de novo* assembly and redundant sequences were removed by the CD-HIT-EST algorithm in CD-HIT software^42^. The non-redundant transcriptome assemblies were supplied into the MAKER pipeline along with *G. max* protein sequences, and the complete protein sequences of *Arabidopsis* from Uniprot as evidence for the homology based gene prediction. For *ab initio* gene prediction, we used AUGUSTUS software^43^ with mungbean (*V. radiata*) training set. The resulting protein sequences were annotated using InterProScan5^44^.

Using the Pfam annotations from InterProScan result, transcription factors (TF) of adzuki bean proteins from *V. angularis* var. *angularis* and *V. nakashimae* were classified based on the TF classification rules described in Lang et al^19^. For comparative analysis of TF contents, we further classified the TF from the protein sequences of 8 plant genomes (*A. thaliana, G. max, M. truncatula, C. cajan, C. arietinum, B. distachyon, Z. mays, O. sativa*).

Using Pfam^45^ HMM profile of LRR domain (PF00560, PF07725, PF12799, PF13504, PF13516, PF13855 and PF14580) and NB-ARC domain (PF00931), we performed a genome-wide scan to find NBS and LRR domain containing proteins. The sequence blocks matched as LRR and NBS domain were re-aligned to construct *V. angularis* var. *angularis* specific hmm profiles of LRR and NBS using the HMMER software package with default parameter for “hmmbuild” command^46^. Using the *V. angularis*-specific hmm profiles, proteins with NBS and/or LRR domain were identified using HMMER software package with default parameter for “hmmscan” command. The putative functions of NBS-LRR were annotated using the blast result against Uniprot database^47^.

We further linked the scaffolds based on the gene order conservation (synteny) of *P. vulgaris*, *V. radiata*, and *V. angularis*. The pairwise synteny relationships, and the Ks values of gene pairs were calculated using MCSCANX software^11^. The synteny blocks between *V. radiata* and *P. vulgaris* were used as links for scaffolding. We retrieved every link that could be deduced from synteny blocks and concatenated each other to build consensus links (Supplementary Fig. S3). The orientation of the scaffold was determined by the orientation of the gene orders of the synteny blocks used for links.

### Genetic map construction

To construct a genetic map of adzuki bean, we genotyped 133 F_4_ lines derived from the cross between Gyeongwon and *V. nakashimae* (IT178530) using genotyping by sequencing (GBS)^12^ (Supplementary Table S23). The genomic DNA of each line was extracted and fragmented by the ApeKI restriction enzyme. Validated PCR fragments using the Agilent Technologies Bioanalyzer 2100 were constructed into GBS sequence library and were sequenced using the Illumina HiSeq 2000. We retrieved the sequences flanked with ApeKI restriction site from *de novo* assembled scaffolds. The short reads were mapped onto the ApeKI flanking sequences using software Bowtie2^13^. The genotypes of 133 RILs were collected from the genomic positions showing over 10 read depth, and we determined homo- and heterozygous genotypes following SAMtools genotyping statistics with default parameter^48^. If the arrays of paternal or maternal genotypes of 133 individuals within 1 Kb are consistent each other we regarded these sites as co-segregating block, and we used one representative site with lowest ‘N' to construct genetic map. The genotypes of 133 F_4_ lines were supplied to JoinMap 4, and we constructed 11 linkage groups using regression mapping algorithm with Kosambi mapping function.

### Resequencing of three wild Vigna species

The sequence reads of *V. nakashimae*, *V. nepalensis*, and *V. angularis* var. *nipponensis* were aligned to our reference genome of *V. angularis* var. *angularis* using the BWA-MEM algorithm of BWA software with default options^25^ (Supplementary Table S23). The resulting read mapping files were supplied to AddOrReplaceReadGroups, MarkDuplicates, FixMateInformation, RealignerTargetCreator, IndelRealigner, BaseRecalibrator, PrintReads modules included in Picard (http://picard.sourceforge.net) and GenomeAnalysisTK (3.1–1 version) to optimize the sequence alignment^49^. As there is no comprehensive set of high-confidence known variant site for adzuki bean genome, we used the parameter –run_without_dbsnp_potentially_ruining_quality for BaseRecalibrator step. For variation calling process, the UnifiedGenotyper module of GenomeAnalysisTK was used. We set the cut-off for the mapping quality as 30 and the sites lower than the cut-off were ignored for further analyses. Moreover, we regarded the genomic positions showing the read depth over twice of the sequence coverage as duplicated region and discarded for accuracy of variation calling. Additionally, if more than four reads with zero mapping quality mapped on certain genomic position or the ratio of the read with zero mapping quality to total mapped reads exceed 10 percent, the genomic position was excluded.

### Species tree construction

We constructed the species tree among the closely related diploid warm season legume species, such as *P. vulgaris*, *V. radiata*, *V. angularis* var. *angularis*, *V. angularis* var. *nipponensis*, *V. nakashimae*, and *V. nepalensis* based on Bayesian Markov Chain Monte Carlo (MCMC) analysis using the 60 orthologous loci by *BEAST of the software package BEAST version 1.8^50^. To find the high-confident ortholog, we used synteny relationship among four legume species such as, *P. vulgaris*, *V. radiata*, *V. angularis*, and *M. truncatula*. Even though we didn't include the *M. truncatula* for species tree construction, we used it to narrow down the confident orthologs. Among the retrieved orthologs, 60 highly conserved ones were chosen, that showed the low relative standard deviation (RSD < 0.00001) of protein length to have clear speciation signal. To this confident ortholog set, we also added the corresponding orthologs of *V. nepalensis*, *V. angularis* var. *nipponensis*, and *V. nakashimae*. The coding sequences of the orthologous loci were aligned using Prank software with –translation option^51^. The starting tree for the analysis was set to random, and we implemented four runs of MCMC with the length of chain 50 million and the parameters logged at every 5000 steps. The substitution model was determined using software ProtTest to choose JTT as best model^52^. The relaxed clock model with log normally distributed uncorrelated rates was used, and the divergence time for each node was calibrated using 8 MYA divergence of *Vigna* and *Phaseolus* of previous estimation^32^.

#### Accession codes

The adzuki bean genome information such as genome assembly, gene prediction and annotation, genetic markers, and other related files of this study can be searched and downloaded from http://plantgenomics.snu.ac.kr. This Whole Genome Shotgun project has been deposited at DDBJ/EMBL/GenBank under the accession JRFV00000000.

## Supplementary Material

Supplementary Information Supplementary information

Supplementary Information Supplementary Table S6

Supplementary Information Supplementary Table S7

Supplementary Information Supplementary Table S9

Supplementary Information Supplementary Table S11

Supplementary Information Supplementary Table S12

Supplementary Information Supplementary Table S13

Supplementary Information Supplementary Table S17

Supplementary Information Supplementary Table S18

Supplementary Information Supplementary Table S19

Supplementary Information Supplementary Table S22
