# Supplementary material for: Draft genome sequence of adzuki bean, Vigna angularis
Source: Sci Rep. 2015 Jan 28;5:8069. doi: 10.1038/srep08069 (PMC5389050; doi:10.1038/srep08069)
Supplement: Supplementary Information [file srep08069-s1.doc]

**Draft genome sequence of adzuki bean, *Vigna angularis***

Yang Jae Kang^1^, Dani Satyawan^1^, Sangrea Shim^1^, Taeyoung Lee^1^, Jayern Lee^1^, Won Joo Hwang^1^, Sue K Kim^1^, Puji Lestari^2^, Kularb Laosatit^3^, Kil Hyun Kim^4^, Tae Joung Ha^5^, Annapurna Chitikineni^6^, Moon Young Kim^1^, Jong-Min Ko^7^, Jae-Gyun Gwag^8^, Jung-Kyung Moon^4^, Yeong-Ho Lee^1^, Beom-Seok Park^9^, Rajeev K Varshney^6^, Suk-Ha Lee^1,10^

^1^Department of Plant Science and Research Institute for Agriculture and Life Sciences, Seoul National University, Seoul 151-921, Korea. ^2^Indonesian Center for Agricultural Biotechnology and Genetic Resources Research and Development (ICABIOGRAD-IAARD), Jalan Tentara Pelajar No. 3A Bogor 16111, Indonesia. ^3^Program in Plant Breeding, Faculty of Agriculture at Kamphaeng Saen, Kasetsart University, Kamphaeng Saen, Nakhon Pathom 73140, Thailand. ^4^National Institute of Crop Science, Rural Development Administration, Suwon, 441-857, Korea. ^5^Research Policy Bureau, R&D Performance Evaluation & Management Division, Nongsaengmyeong-ro 300, Wansan-gu, Junju, 560-500, Korea. ^6^International Crops Research Institute for the Semi-Arid Tropics, Patancheru, Andhra Pradesh, India. ^7^Soybean Research Team, Legume & Oil Crop Research Division, Jeompiljae-ro 20, Miryang, Gyeongnamdo, 627-803, Korea. ^8^National Agrobiodiversity Center of NAAS, RDA, Suwon 441-707, Korea. ^9^Agricultural Genome Center, National Academy of Agricultural Science, Rural Development Administration, Suwon, 441-707, Korea. ^10^Plant Genomics and Breeding Institute, Seoul National University, Seoul, 151-921, Korea.

Correspondence should be addressed to Suk-Ha Lee ([sukhalee@snu.ac.kr](mailto:sukhalee@snu.ac.kr)).


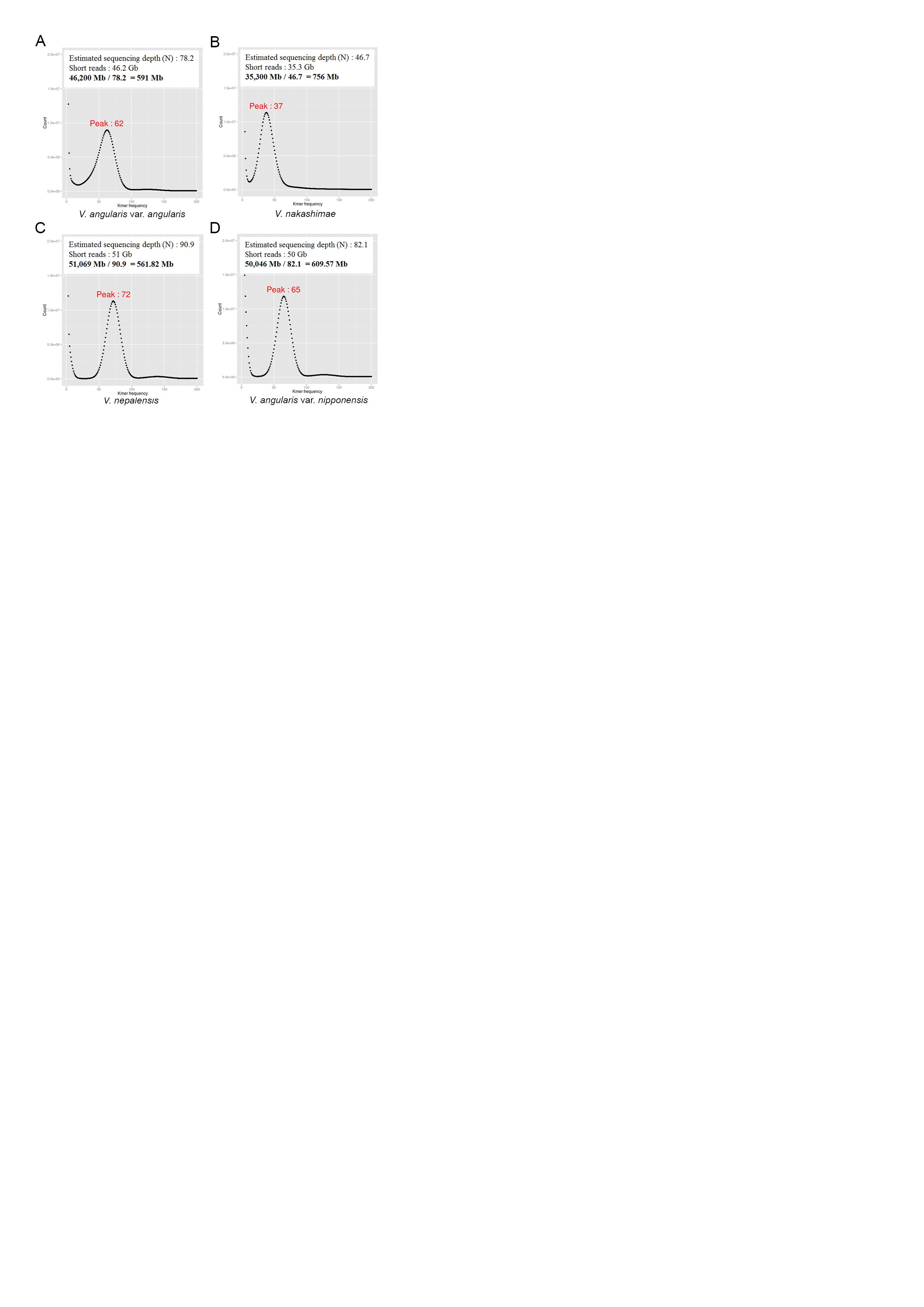


**Supplementary Figure S1**. **Kmer frequency analysis to estimate the genome sizes using the paired-end short reads.** The distributions of Kmer frequency of Illumina Hiseq short read sequences were plotted. A: *V. angularis* var. *angularis*, B: *V. nakashimae*, C: *V. nepalensis*, and D: *V. angularis* var. *nipponensis*.


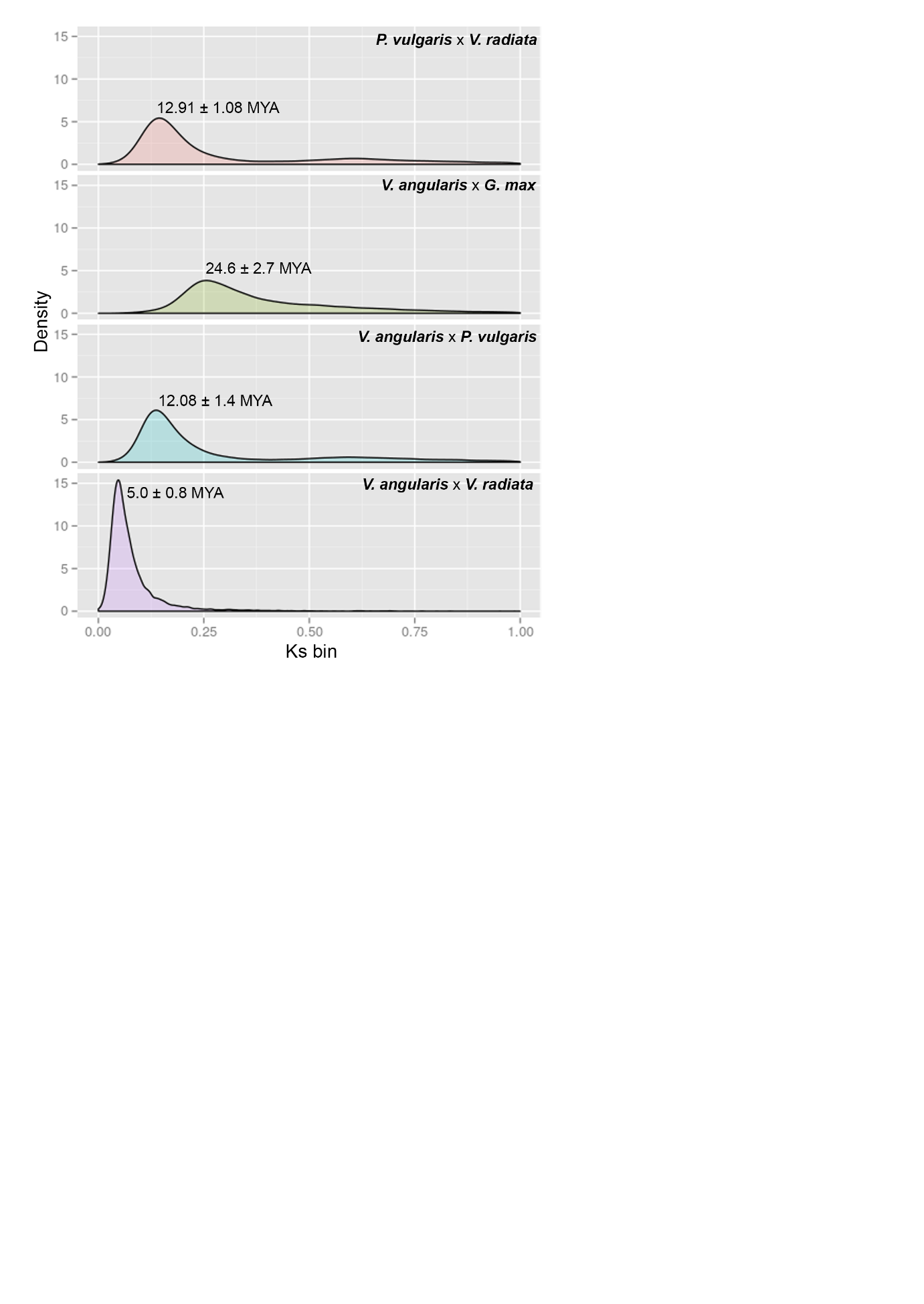


**Supplementary Figure S2.** **The divergence times between two species.** The divergence times were estimated using the mean Ks frequency of gene pairs within synteny blocks between *V.angularis* and each closely related legume genome. For MYA conversion, the substitution rate, 6.1 x 10^-9^, was used.


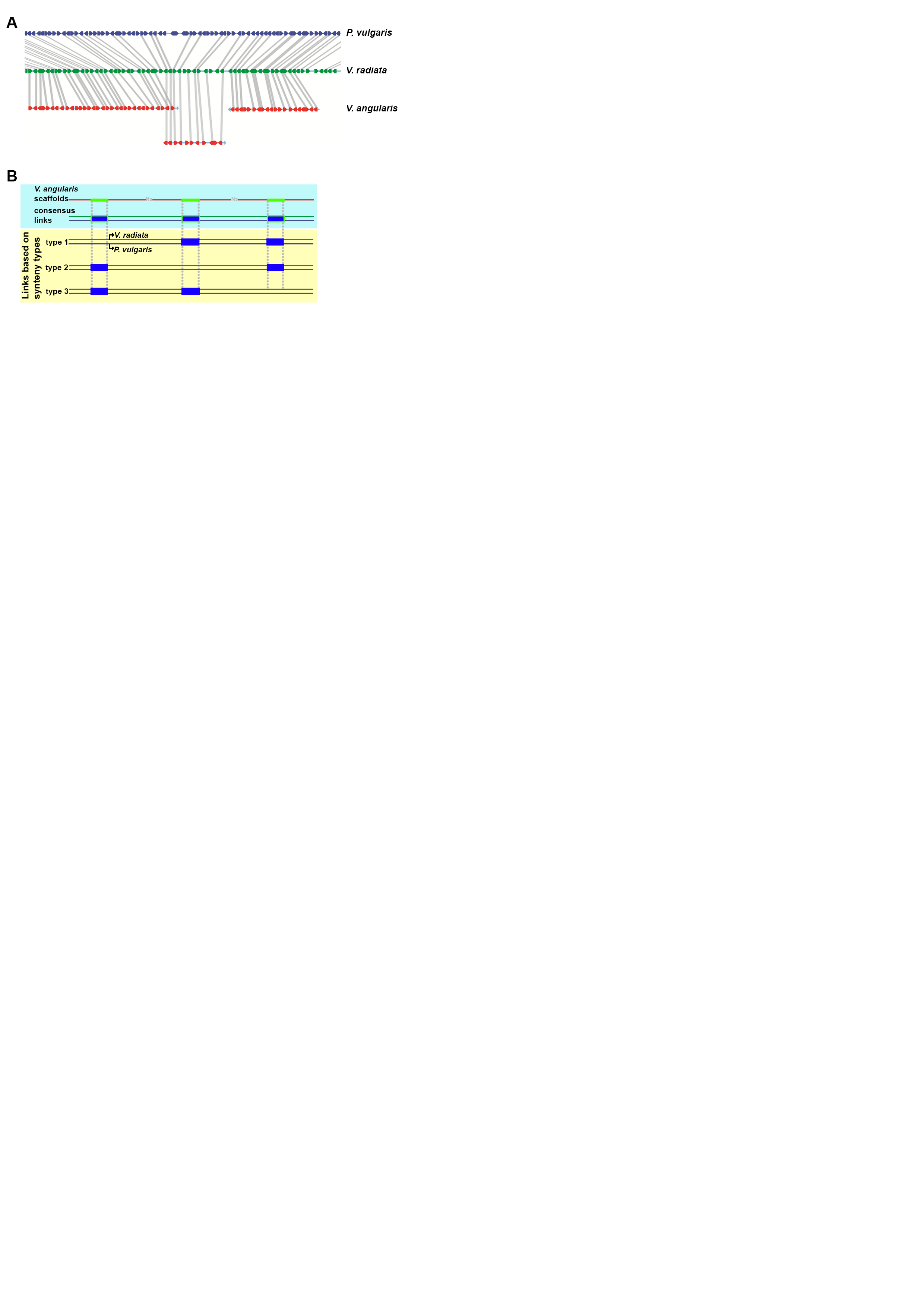


**Supplementary Figure S3**. **Synteny-based scaffolding method**. A: An example of synteny blocks of *P. vulgaris*, *V. radiata*, and *V. angularis* supporting the links among *V. angularis* scaffolds. B: Schematic illustration of synteny-based scaffolding using the consensus links from types of synte ny blocks.


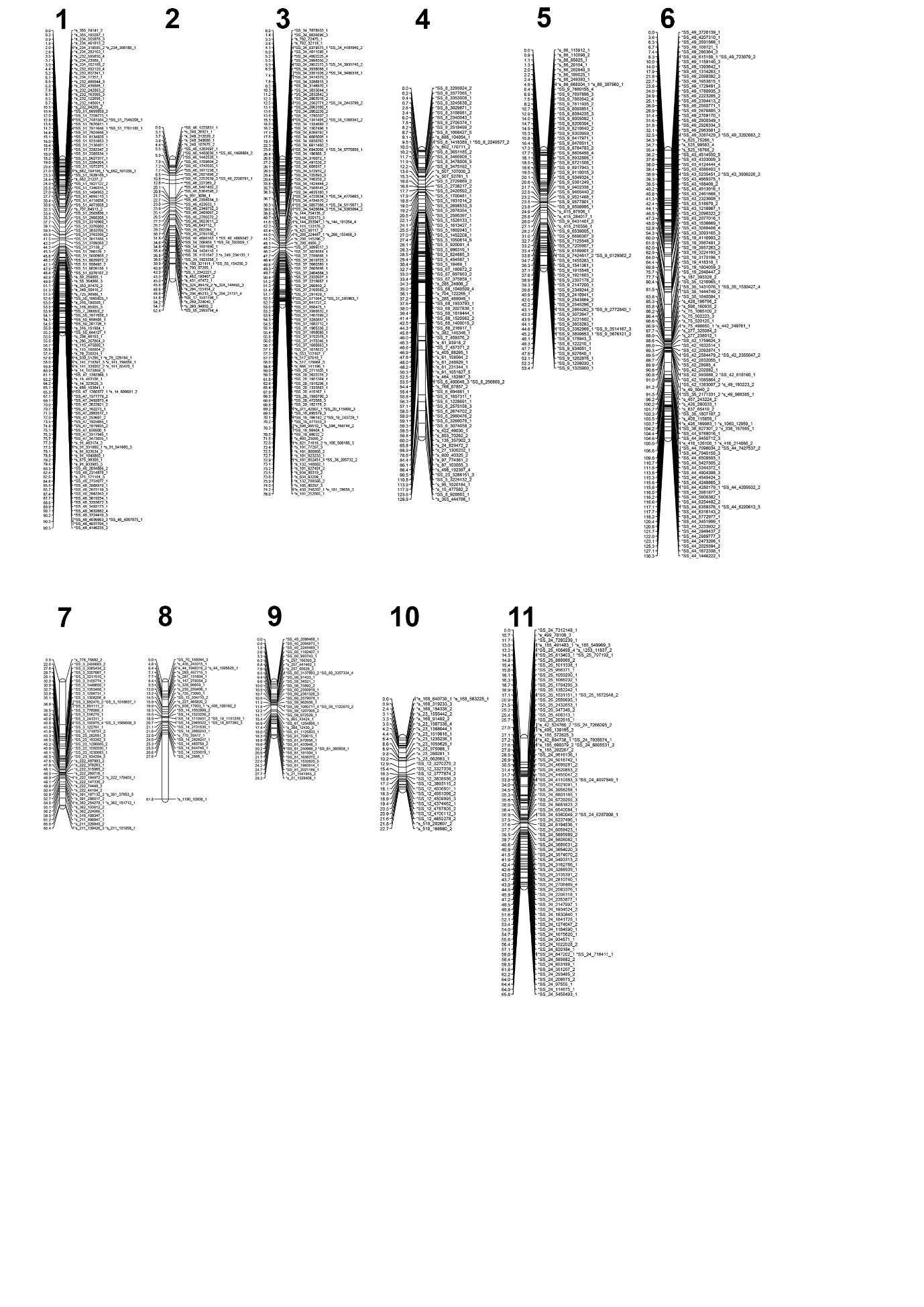


**Supplementary Figure S4**. **The genetic map of adzuki bean.** The genetic map was constructed by GBS method on 133 F4 RILs derived from the cross between *V. angularis* var. *angularis* and *V. nakashimae*. Prefix of ‘s’ stands for scaffolds, and prefix of ‘SS’ for super-scaffolds.


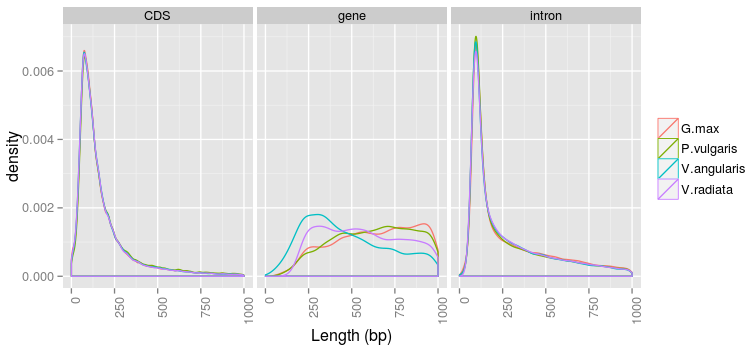


**Supplementary Figure S5**. **Density plot of the intron, CDS and gene length of *G. max*, *P. vulgaris*, *V. radiata*, and *V. angularis* var. *angularis*.**


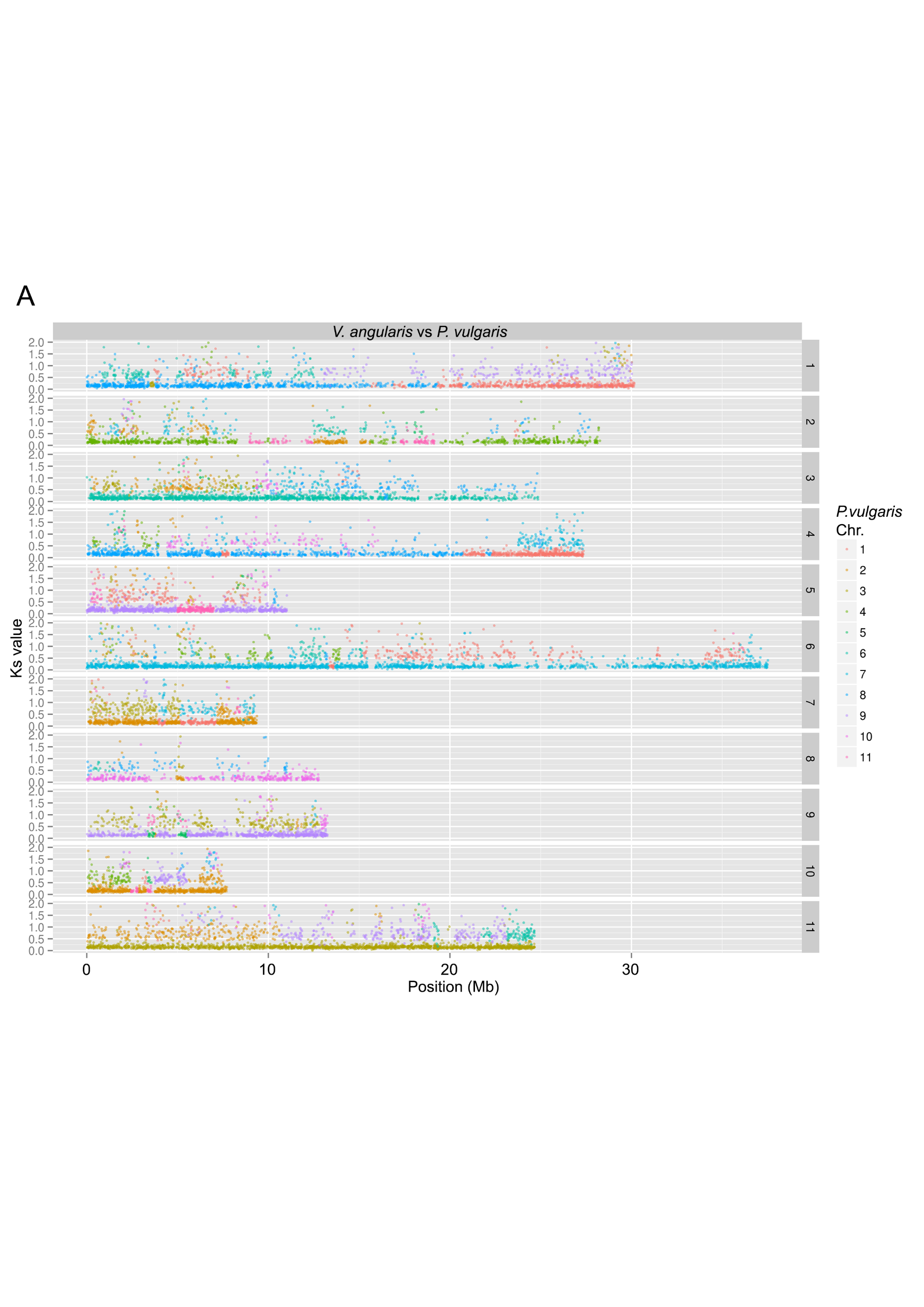


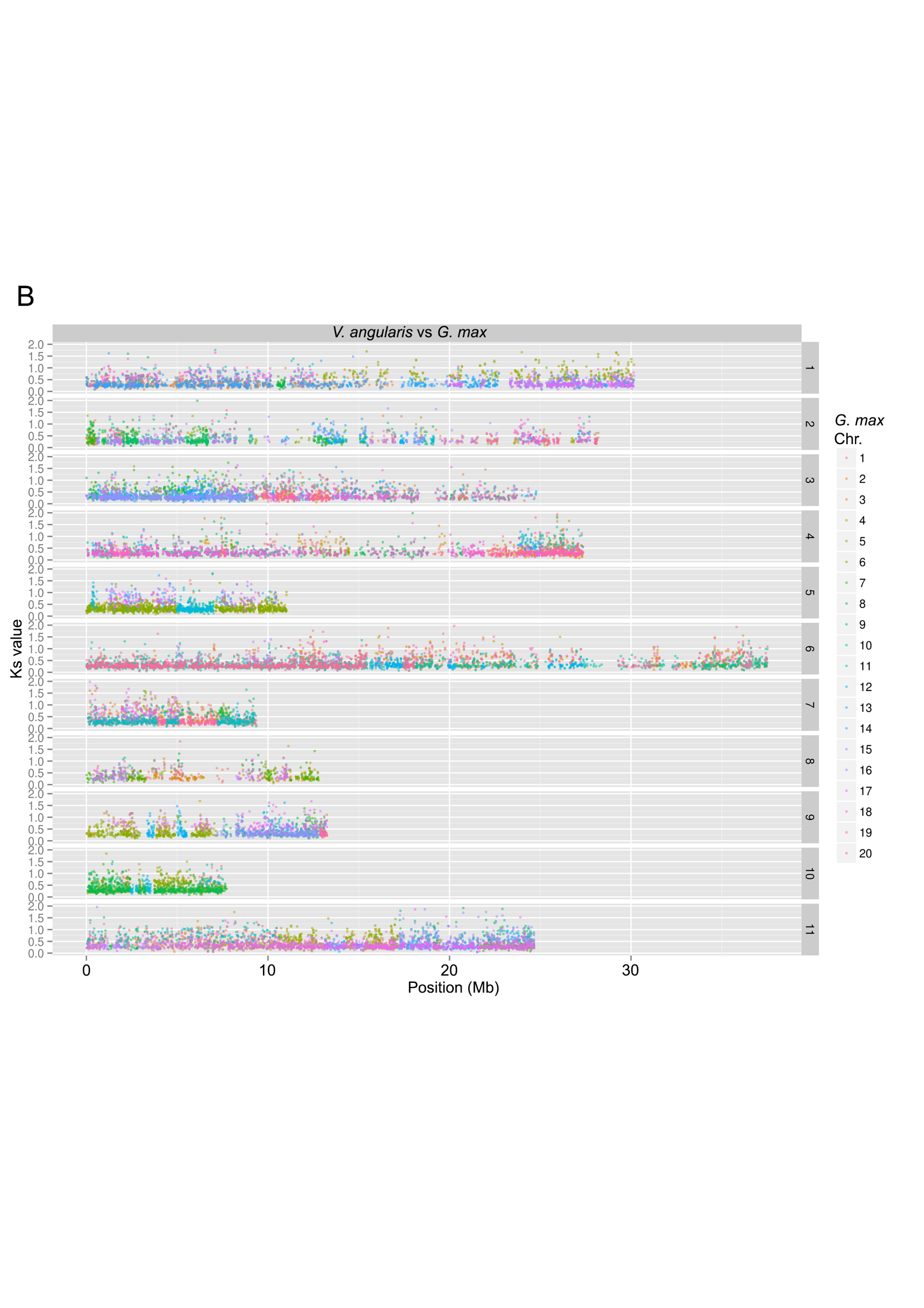


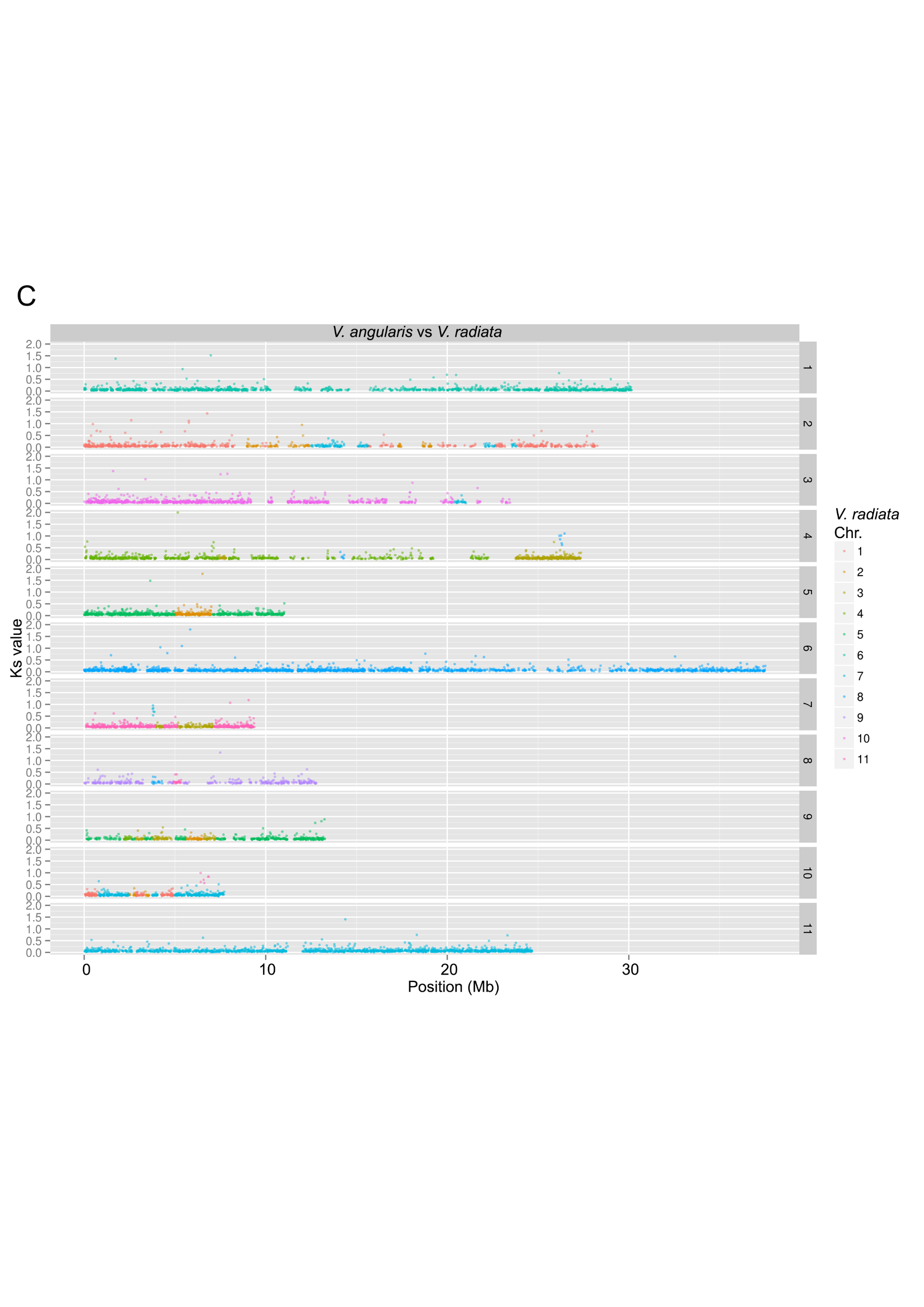


**Supplementary Figure S6.** **Enlarged visualization of the synteny relationships among *V. angularis*, *V. radiata*, *P. vulgaris* and *G. max*.** The x-axis indicates chromosomal locations of genes in synteny relationship, the y-axis indicates Ks value of the corresponding gene pair, number in gray box of right end of plot indicates the chromosome number of *V. angularis*. The color of dot indicates the chromosome of each target genome showing both conservation of gene order as well as chromosomal rearrangements of synteny blocks. A. *V. angularis* vs *P. vulgaris*, B. *V. angularis* vs *G. max*, and C. *V. angularis* vs *V. radiata*


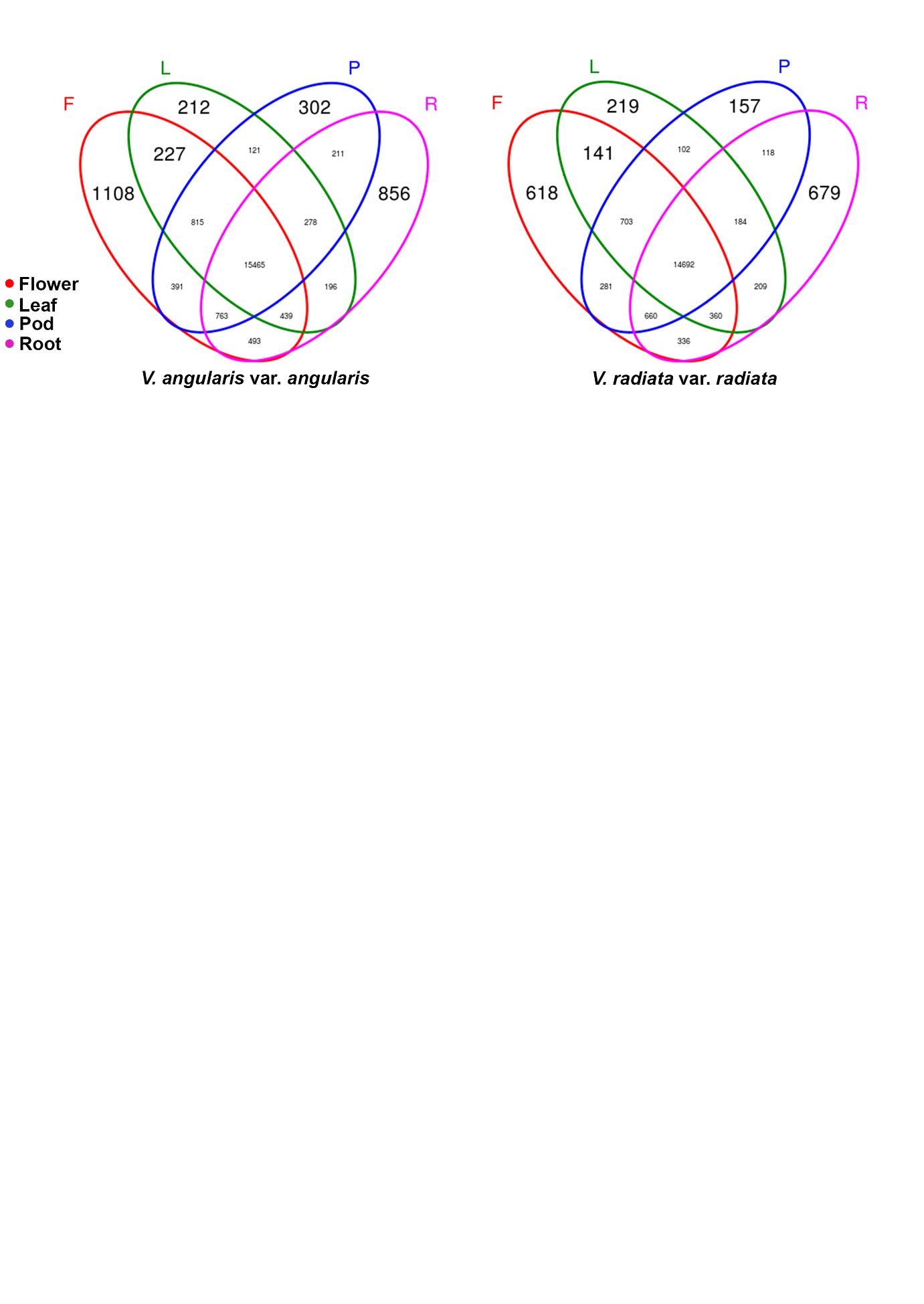


**Supplementary Figure S7**. **Classification of gene expression by tissue specificity of flower, leaf, pod and root for *V. angularis* var. *angularis* and *V. radiata* var. *radiata*.**


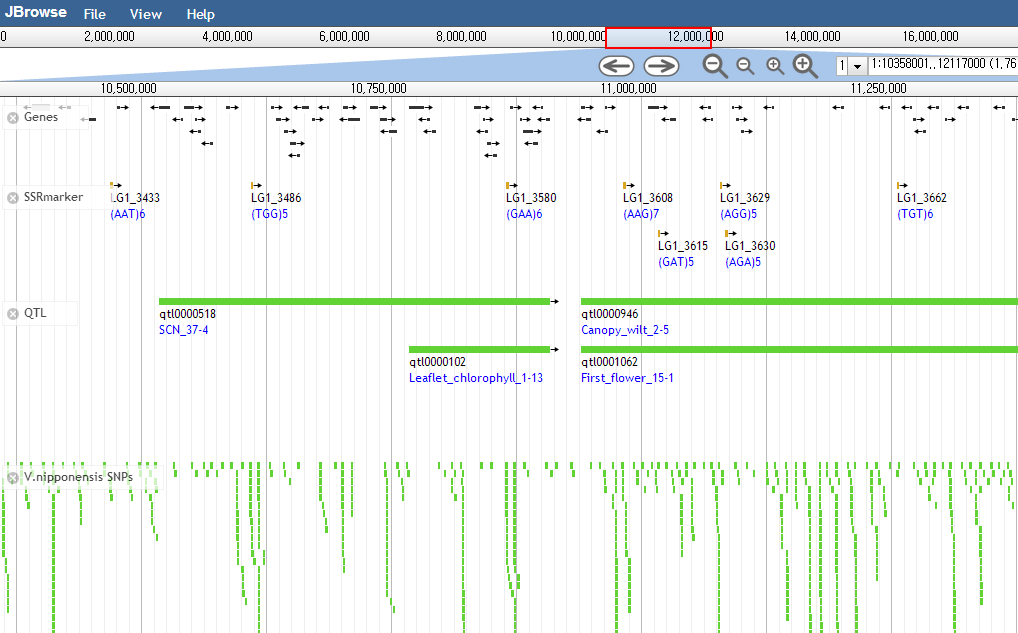


**Supplementary Figure S8.** **Example of *V. angularis* database.** The website was constructed based on Jbrowse containing the gene, SSR, SNP, and predicted QTL regions. This browser is accessible through <http://plantgenomics.snu.ac.kr/>.


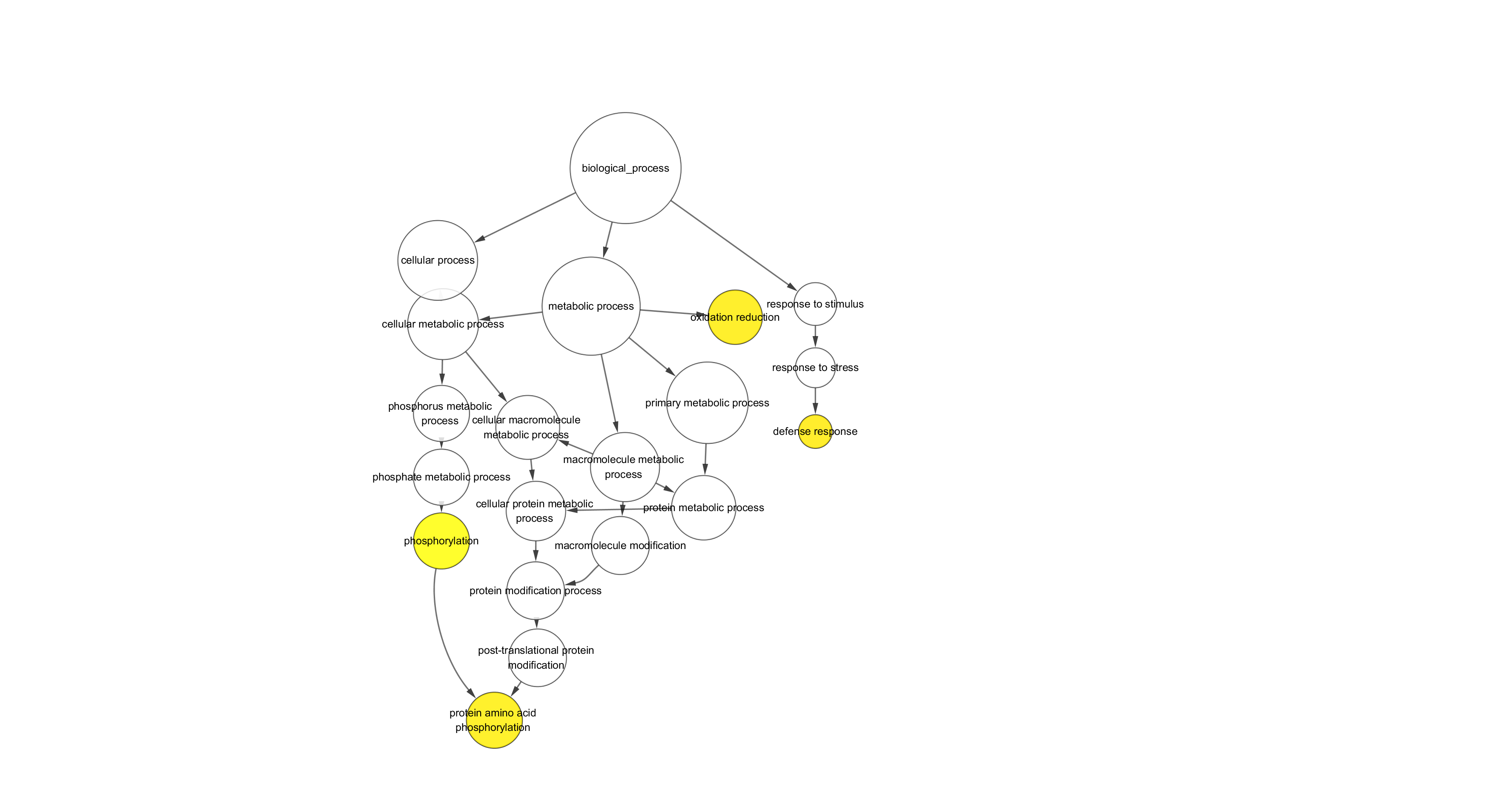


**Supplementary Figure S9.** **Gene ontology enrichment analysis with bonferroni Family-Wise Error Rate correction on the tandemly duplicated genes within the genome of *V. angularis* var. *angularis.*** Significantly enriched GO terms are highlighted by yellow color.

| **Supplementary Table S1**. Flowcytometry result of the leaf sample of *V. angularis* var. *angularis* | | | | |
| --- | --- | --- | --- | --- |
| Sample name | Sample | Standard(int.) | DNA content | St. Dev |
|  | G0+G1 | G0+G1 |  |  |
|  | mean | mean | (pg/2C) | ± |
| Adzuki bean |  | CEN |  |  |
|  | 144.74 | 287.87 | 1.26 |  |
|  | 155.8 | 312.92 | 1.24 |  |
|  | 168.86 | 337.79 | 1.25 |  |
|  | 181.22 | 358.52 | 1.26 |  |
|  | 194.35 | 389.47 | 1.25 |  |
|  | 209.59 | 423.12 | 1.24 |  |
|  | 224.54 | 452.84 | 1.24 |  |
|  | 241.3 | 484.21 | 1.25 |  |
|  | 259.04 | 517.3 | 1.25 |  |
|  | 276.61 | 552.06 | 1.25 |  |
|  | 297.42 | 595.43 | 1.25 |  |
|  | 319.67 | 638.54 | 1.25 |  |
|  |  |  | **1.25** | **0.007** |
| 1.0 pg= 980 Mbp |  | *Mbp* | *612.15* | *3.43* |

| **Supplementary Table S2**. Summary of the sequencing information for adzuki bean genome analysis | | | | | | | | |
| --- | --- | --- | --- | --- | --- | --- | --- | --- |
| Purpose | Acc no | Common name | Scientific name | Platform | Library | Total reads | Total bases | Sequencing depth^*^ |
| Genome assembly | IT213134 | Gyeongwon | *V. angularis* var*. angularis* | Illumina Hiseq | Fragment (1) | 458,328,288 | 46,291,157,088 | 78.33 |
|  |  |  |  |  | Fragment (2) | 442,913,006 | 44,734,213,606 | 75.69 |
|  |  |  |  |  | Mate-pair 5k (1) | 273,827,622 | 27,656,589,822 | 46.80 |
|  |  |  |  |  | Mate-pair 5k (2) | 287,592,844 | 29,046,877,244 | 49.15 |
|  |  |  |  |  | Mate-pair 10k | 234,913,474 | 23,726,260,874 | 40.15 |
|  |  |  |  | GS-FLX plus | Single linear | 1,288,626 | 590,023,578 | 1.00 |
| Resequencing | IT241912 | - | *V. angularis* var*. nipponnesis* | Illumina Hiseq | Paired-end | 426,560,794 | 50,046,567,994 | 84.68 |
|  | AusTRCF85148 | - | *V. nepalensis* | Illumina Hiseq | Paired-end | 433,501,676 | 51,069,013,176 | 86.41 |
|  | IT178530 ^b^ | - | *V. nakashimae* | Illumina Hiseq | Paired-end | 349,772,128 | 35,326,984,928 | 59.77 |
| a. Sequencing depths were determined by genome size estimation (591 Mb) based on kmer analysis | | | | | | | | |
| b. Lestari et al. 2014 | |  |  |  |  |  |  |  |

| **Supplementary Table S3**. The coverage report for *de novo* assembly of each sequencing library | | | | |
| --- | --- | --- | --- | --- |
| Library type | Library name | Insert size estimation from assembly (bp) | Number of reads | Percent of  used reads (%) |
| Fragment | GSFLX pseudo fragment lib. | -25±20 | 64,350 | 81.7 |
|  | Illumina_180 | -32±20 | 458,328,288 | 73.7 |
|  | Illumina_180_1 | -26±20 | 442,913,006 | 72.5 |
| Jumping | Illumina_10k | 7206±1803 | 234,913,474 | 14.7 |
|  | Illumina_5k | 5032±547 | 273,827,622 | 29.5 |
|  | Illumina_5k_1 | 5407±571 | 287,592,844 | 42.3 |

| **Supplementary Table S4**. Summary of genome assemblies | | |  |
| --- | --- | --- | --- |
|  | *Vigna angularis* | *Vigna nakashimae* | *Vigna radiata* var. *radiata* |
| **Genome** |  |  |  |
| Chromosomes | 2n=2x=22 | 2n=2x=22 | 2n = 2x = 22 |
| Genome size ^a^ | 590,792,800 | 756,466,486 | 548,078,290 |
| **Genome assembly** |  |  |  |
| Minimum contig size | 1,000 | 1,000 | 1,000 |
| Number of contigs | 36,516 | 59,809 | 25,922 |
| Number of contigs per Mb | 82.3 | 146.7 | 56 |
| Number of scaffolds | 3,883 | - | 2,748 |
| Total scaffold length, with gaps (bp) | 443,436,934 | 431,029,034 | 463,143,055 |
| N50 contig size (kb) | 21.9 | 12.2 | 41.8 |
| N50 scaffold size (kb) | 703 | - | 1,516 |
| Number of scaffolds per Mb | 8.76 | - | 5.93 |
| GC contents (%) | 36 | 37 | 33.3 |
| **Genetic map anchoring** |  |  |  |
| Number of linkage groups | 11 |  | 11 |
| Number of anchored scaffolds with orientation | 80 |  | 153 |
| Number of anchored scaffolds without orientation | 78 |  | 86 |
| N50 pseudo-molecule size (mb) | 25 |  | 35.4 |
| **Gene prediction and annotations** |  |  |  |
| Number of high-confident genes | 26,857 | 23,197 ^b^ | 22,427 |
| Number of interpro annotated genes | 21,532 | 19,745 | 19,704 |
| 248 Core eukaryotic genes (%) with BLASTP | 99 | 97 | 97 |
| Complete (%) to CEGs by CEGMA pipeline | 86.29 |  |  |
| Partial (%) to CEGs by CEGMA pipeline | 95.97 |  |  |
| a. Genome sizes were estimated by 25-kmer frequency distribution | |  |  |
| b. Gene prediction on the contigs over 10 Kb |  |  |  |

| **Supplementary Table S5.** Comparison of genome assemblies of *V. angularis* var. *angularis* using different sizes of libraries | | |
| --- | --- | --- |
|  | Libraries and methods | |
|  | Fragment | Fragment |
|  | Mate pair (5k, 10k) | Mate pair (5k, 10k) |
|  | Pseudo mate (5k)* | Pseudo mate (5k)* |
|  | ALLPATH-LG | ALLPATH-LG +  Synteny based scaffolding |
| Number of scaffolds | 3,883 | 3,641 |
| N50 scaffold size in kb | 704 | 1,530 |
| N90 scaffold size in kb | 122 | 127 |
| Maximum length of scaffold in kb | 4,431 | 11,114 |
| Total scaffolds length without gap in Mb | 399 | 399 |
| * Pseudo mate pairs were derived from GS-FLX assembly | | |

| **Supplementary Table S8.** Summary of the anchoring the scaffolds onto GBS-based genetic map | | |
| --- | --- | --- |
|  |  | GBS-based genetic map |
| Pseudo-molecule only | Total length (bp) | 209,972,228 |
|  | Number of contig or scaffold | 11 |
|  | Maximum length of contigs or scaffolds (bp) | 34,932,637 |
|  | Minimum length of contigs or scaffolds (bp) | 7,241,346 |
|  | N90 (bp) | 10,538,746 |
|  | N50 | 25,092,484 |
|  | N10 | 34,932,637 |
| Pseudo-molecule + scaffolds | Total length (bp) | 399,301,794 |
|  | Number of contig or scaffold | 3,494 |
|  | Maximum length of contigs or scaffolds | 34,932,637 |
|  | Minimum length of contigs or scaffolds | 941 |
|  | N90 | 132,555 |
|  | N50 | 8,988,578 |
|  | N10 | 27,846,940 |

| **Supplementary Table S10.** Statistics of the transcriptome assembly of *V. angularis* var. *angularis* | | | | |
| --- | --- | --- | --- | --- |
|  | Flower | Root | Pod | Leaf |
| Read counts | 36,112,812 | 42,697,406 | 51,071,240 | 39,813,160 |
| Total bases (bp) | 3,647,394,012 | 4,312,438,006 | 5,158,195,240 | 4,021,129,160 |
| Number of contigs over 200 bp | 142,534 | 167,870 | 104,496 | 145,023 |
| Number of contigs over N50 | 26,093 | 32,890 | 18,853 | 28,466 |
| N50 (bp) | 869 | 1,884 | 1,415 | 1,714 |
| Maximum length of contigs (bp) | 25,997 | 17,294 | 24,573 | 17,647 |
| Coverage length of contigs over 200 bp | 89,600,000 | 192,400,000 | 91,850,000 | 153,000,000 |
| Number of total predicted protein | 41,530 | 95,730 | 52,228 | 83,301 |
| Number of complete protein | 10,351 | 62,922 | 21,825 | 49,509 |

| **Supplementary Table S14**. Summary of transposable elements in cultivated (*V. angularis* var. *angularis*) adzuki bean genome | | | | | |
| --- | --- | --- | --- | --- | --- |
|  |  | Length (bp) | Number of elements | Percent in repeat | Percent in genome |
| **Class I elements**  **(Retroelements)** | | |  |  |  |
| LTR Retrotransposons | | |  |  |  |
|  | LTR/Gypsy | 75,796,525 | 82,273 | 44.06 | 18.98 |
|  | LTR/Copia | 39,757,365 | 50,530 | 23.11 | 9.96 |
|  | LTR/Others | 43,290,910 | 53,891 | 25.16 | 10.84 |
| Non-LTR Retrotransposons | | |  |  |  |
|  | LINE/Others | 23,869 | 56 | 0.01 | 0.01 |
|  | LINE/Ukn | 3,407 | 5 | 0.00 | 0.00 |
| **Class II elements**  **(DNA Transposons)** | | |  |  |  |
|  | CACTA | 3,027,231 | 3,651 | 1.76 | 0.76 |
|  | Mutator | 5,056,012 | 7,485 | 2.94 | 1.27 |
|  | PIF-Harbinge | 1,212,422 | 1,251 | 0.70 | 0.30 |
|  | hAT | 109,362 | 107 | 0.06 | 0.03 |
|  | Helitron | 99,349 | 160 | 0.06 | 0.02 |
|  | MULE-MuDR | 827,857 | 780 | 0.48 | 0.21 |
|  | Tc1-Mariner | 349,875 | 501 | 0.20 | 0.09 |
| Low_complexity | | 2,077,077 | 36,134 | 1.21 | 0.52 |
| Simple_repeat | | 7,038,465 | 135,947 | 4.09 | 1.76 |
| **Total** |  | 172,048,010 |  |  | 43.09 |

| **Supplementary Table S15**. Summary of resequencing of wild adzuki bean accessions | | | | | | | | | |
| --- | --- | --- | --- | --- | --- | --- | --- | --- | --- |
| Cultivar | Accession Number | Genome Size (Mb) | Raw sequencing data | | | Aligned sequencing data | | | |
|  |  |  | No. of reads | Total No. of bases sequenced | Genome fold coverage | No. of reads | No. of reads(%) | Total No. of bases mapped | Genome fold coverage |
| *V. nakashimae* | IT178530 | 756 | 358,634,101 | 36,222,044,201 | 47.91 | 230,832,583 | 64.36 | 23,314,090,883 | 30.84 |
| *V. nepalensis* | AusTRCF85148 | 562 | 445,393,049 | 44,984,697,949 | 80.07 | 389,389,491 | 87.43 | 39,328,338,591 | 70.00 |
| *V. nipponensis* | IT241912 | 610 | 434,762,606 | 43,911,023,206 | 72.04 | 365,581,530 | 84.09 | 36,923,734,530 | 60.57 |

| **Supplementary Table S16**. Variation summary resulted from whole genome resequencing of wild adzuki beans | | | | | | | | | | | | | | |
| --- | --- | --- | --- | --- | --- | --- | --- | --- | --- | --- | --- | --- | --- | --- |
| Cultivar | Accession number | No. of mapped  position | Variant type | Total no. of variation | Variation frequency (No. of SNPs per Kb) | Non-genic variant | Genic (No. of genes) | | | | | | | |
|  |  |  |  |  |  |  | Total | 5' UTR | Coding | | | | 3' UTR | Intron |
|  |  |  |  |  |  |  |  |  | Synonymous | Non-synonymous | Frameshift | Inframe |  |  |
| *V. nakashimae* | IT178530 | 237,755,346 | SNP | 3,342,795 | 14.06 | 2,717,939 | 624,856 (21,878) | 9,002 (4,641) | 12,280 (6,630) | 21,296 (9,184) | - | - | 22,805 (9,060) | 562,751 (19,505) |
|  |  |  | Indel | 11 | 0.00 | 8 | 3 (3) | 0 (0) | - | - | 0 (0) | 0 (0) | 1 (1) | 2 (2) |
| *V. nepalensis* | AusTRCF85148 | 342,908,304 | SNP | 3,511,378 | 10.24 | 3,078,168 | 433,210 (20,740) | 5,226 (2,855) | 9,332 (4,460) | 18,034 (6,464) | - | - | 12,229 (5,449) | 390,163 (18,309) |
|  |  |  | Indel | 481,569 | 1.40 | 410,232 | 71,337 (14,956) | 1,437 (1,199) | - | - | 638 (549) | 458 (427) | 3,315 (2,568) | 65,800 (13,823) |
| *V. angularis* var. *nipponensis* | IT241912 | 366,387,857 | SNP | 667,097 | 1.82 | 591,621 | 75,476 (9,471) | 907 (488) | 1,917 (841) | 3,840 (1,421) | - | - | 1,895 (911) | 67,177 (8,319) |
|  |  |  | Indel | 97,932 | 0.27 | 83,899 | 14,033 (5,828) | 273 (246) | - | - | 129 (116) | 94 (89) | 568 (475) | 13,028 (5,390) |

| **Supplementary Table S20**. List of NBS-LRR proteins and the Uniprot annotation | | | |
| --- | --- | --- | --- |
| Genename | Domain | Uniprot ID | Descrition |
| Vang11g13870.1 | NBS-LRR | sp|Q40392|TMVRN_NICGU | TMV resistance protein N |
| Vang11g03590.1 | NBS-LRR | sp|Q9FKZ1|DRL42_ARATH | Probable disease resistance protein At5g66900 |
| Vang11g03570.1 | NBS-LRR | sp|Q9FKZ1|DRL42_ARATH | Probable disease resistance protein At5g66900 |
| Vang11g03500.1 | NBS-LRR | sp|Q9FKZ1|DRL42_ARATH | Probable disease resistance protein At5g66900 |
| Vang10g00850.1 | NBS-LRR | sp|Q40392|TMVRN_NICGU | TMV resistance protein N |
| Vang10g00830.1 | NBS-LRR | sp|Q40392|TMVRN_NICGU | TMV resistance protein N |
| Vang1009s00010.1 | NBS-LRR | sp|Q39214|RPM1_ARATH | Disease resistance protein RPM1 |
| Vang09g07260.1 | NBS-LRR | sp|Q40392|TMVRN_NICGU | TMV resistance protein N |
| Vang09g06870.1 | NBS-LRR | sp|Q9SX38|DRL4_ARATH | Putative disease resistance protein At1g50180 |
| Vang0984s00030.1 | NBS-LRR | sp|Q39214|RPM1_ARATH | Disease resistance protein RPM1 |
| Vang0984s00020.1 | NBS-LRR | sp|Q39214|RPM1_ARATH | Disease resistance protein RPM1 |
| Vang0911s00020.1 | NBS-LRR | sp|Q40392|TMVRN_NICGU | TMV resistance protein N |
| Vang08g04630.1 | NBS-LRR | sp|Q40392|TMVRN_NICGU | TMV resistance protein N |
| Vang08g02100.1 | NBS-LRR | sp|Q9LRR4|R13L1_ARATH | Putative disease resistance RPP13-like protein 1 |
| Vang08g01230.1 | NBS-LRR | sp|O23530|SNC1_ARATH | Protein SUPPRESSOR OF npr1-1, CONSTITUTIVE 1 |
| Vang08g00970.1 | NBS-LRR | sp|Q9T048|DRL27_ARATH | Disease resistance protein At4g27190 |
| Vang08g00920.1 | NBS-LRR | sp|Q9T048|DRL27_ARATH | Disease resistance protein At4g27190 |
| Vang08g00840.1 | NBS-LRR | sp|Q9T048|DRL27_ARATH | Disease resistance protein At4g27190 |
| Vang08g00650.1 | NBS-LRR | sp|Q9T048|DRL27_ARATH | Disease resistance protein At4g27190 |
| Vang0870s00010.1 | NBS-LRR | sp|Q39214|RPM1_ARATH | Disease resistance protein RPM1 |
| Vang0821s00010.1 | NBS-LRR | sp|Q40392|TMVRN_NICGU | TMV resistance protein N |
| Vang07g00820.1 | NBS-LRR | sp|Q8W4J9|RPP8_ARATH | Disease resistance protein RPP8 |
| Vang07g00560.1 | NBS-LRR | sp|Q9FKZ1|DRL42_ARATH | Probable disease resistance protein At5g66900 |
| Vang0747s00020.1 | NBS-LRR | sp|O81825|DRL28_ARATH | Probable disease resistance protein At4g27220 |
| Vang06g21880.1 | NBS-LRR | sp|Q9LRR4|R13L1_ARATH | Putative disease resistance RPP13-like protein 1 |
| Vang06g20640.1 | NBS-LRR | sp|Q7XBQ9|RGA2_SOLBU | Disease resistance protein RGA2 |
| Vang0698s00020.1 | NBS-LRR | sp|O81825|DRL28_ARATH | Probable disease resistance protein At4g27220 |
| Vang0693s00030.1 | NBS-LRR | sp|Q40392|TMVRN_NICGU | TMV resistance protein N |
| Vang0663s00020.1 | NBS-LRR | sp|P60838|DRL1_ARATH | Probable disease resistance protein At1g12280 |
| Vang0663s00010.1 | NBS-LRR | sp|P60838|DRL1_ARATH | Probable disease resistance protein At1g12280 |
| Vang0570s00020.1 | NBS-LRR | sp|Q40392|TMVRN_NICGU | TMV resistance protein N |
| Vang0564s00010.1 | NBS-LRR | sp|Q9LRR4|R13L1_ARATH | Putative disease resistance RPP13-like protein 1 |
| Vang0537s00090.1 | NBS-LRR | sp|Q40392|TMVRN_NICGU | TMV resistance protein N |
| Vang04g06570.1 | NBS-LRR | sp|Q9T048|DRL27_ARATH | Disease resistance protein At4g27190 |
| Vang04g06010.1 | NBS-LRR | sp|O81825|DRL28_ARATH | Probable disease resistance protein At4g27220 |
| Vang04g03590.1 | NBS-LRR | sp|Q9C8T9|DRL19_ARATH | Putative disease resistance protein At1g63350 |
| Vang04g03010.1 | NBS-LRR | sp|Q9SX38|DRL4_ARATH | Putative disease resistance protein At1g50180 |
| Vang04g02230.1 | NBS-LRR | sp|Q9STE7|R13L3_ARATH | Putative disease resistance RPP13-like protein 3 |
| Vang04g01790.1 | NBS-LRR | sp|Q9M667|RPP13_ARATH | Disease resistance protein RPP13 |
| Vang03g18140.1 | NBS-LRR | sp|Q9LRR4|R13L1_ARATH | Putative disease resistance RPP13-like protein 1 |
| Vang03g16290.1 | NBS-LRR | sp|Q9T048|DRL27_ARATH | Disease resistance protein At4g27190 |
| Vang03g15210.1 | NBS-LRR | sp|Q39214|RPM1_ARATH | Disease resistance protein RPM1 |
| Vang03g15160.1 | NBS-LRR | sp|Q39214|RPM1_ARATH | Disease resistance protein RPM1 |
| Vang0335s00100.1 | NBS-LRR | sp|Q9LRR4|R13L1_ARATH | Putative disease resistance RPP13-like protein 1 |
| Vang0306s00030.1 | NBS-LRR | sp|Q9LRR4|R13L1_ARATH | Putative disease resistance RPP13-like protein 1 |
| Vang0304s00040.1 | NBS-LRR | sp|Q40392|TMVRN_NICGU | TMV resistance protein N |
| Vang0304s00010.1 | NBS-LRR | sp|Q9FL92|WRK16_ARATH | Probable WRKY transcription factor 16 |
| Vang02g14420.1 | NBS-LRR | sp|Q9LRR4|R13L1_ARATH | Putative disease resistance RPP13-like protein 1 |
| Vang02g13290.1 | NBS-LRR | sp|O81825|DRL28_ARATH | Probable disease resistance protein At4g27220 |
| Vang02g12360.1 | NBS-LRR | sp|Q40392|TMVRN_NICGU | TMV resistance protein N |
| Vang02g09720.1 | NBS-LRR | sp|Q40392|TMVRN_NICGU | TMV resistance protein N |
| Vang02g03190.1 | NBS-LRR | sp|Q40392|TMVRN_NICGU | TMV resistance protein N |
| Vang0291s00070.1 | NBS-LRR | sp|Q9LRR4|R13L1_ARATH | Putative disease resistance RPP13-like protein 1 |
| Vang0279s00180.1 | NBS-LRR | sp|Q7XBQ9|RGA2_SOLBU | Disease resistance protein RGA2 |
| Vang0250s00070.1 | NBS-LRR | sp|Q40392|TMVRN_NICGU | TMV resistance protein N |
| Vang0248s00060.1 | NBS-LRR | sp|Q40392|TMVRN_NICGU | TMV resistance protein N |
| Vang0229s00130.1 | NBS-LRR | sp|O81825|DRL28_ARATH | Probable disease resistance protein At4g27220 |
| Vang01g19280.1 | NBS-LRR | sp|Q9SZA7|DRL29_ARATH | Probable disease resistance protein At4g33300 |
| Vang01g07720.1 | NBS-LRR | sp|Q8NEE6|FXL13_HUMAN | F-box/LRR-repeat protein 13 |
| Vang01g01600.1 | NBS-LRR | sp|Q9FL92|WRK16_ARATH | Probable WRKY transcription factor 16 |
| Vang0198s00320.1 | NBS-LRR | sp|Q40392|TMVRN_NICGU | TMV resistance protein N |
| Vang0198s00270.1 | NBS-LRR | sp|Q40392|TMVRN_NICGU | TMV resistance protein N |
| Vang0197s00010.1 | NBS-LRR | sp|Q9T048|DRL27_ARATH | Disease resistance protein At4g27190 |
| Vang0162s00090.1 | NBS-LRR | sp|O81825|DRL28_ARATH | Probable disease resistance protein At4g27220 |
| Vang0137s00140.1 | NBS-LRR | sp|Q40392|TMVRN_NICGU | TMV resistance protein N |
| Vang0137s00090.1 | NBS-LRR | sp|Q40392|TMVRN_NICGU | TMV resistance protein N |
| Vang0114s00480.1 | NBS-LRR | sp|Q39214|RPM1_ARATH | Disease resistance protein RPM1 |
| Vang0114s00280.1 | NBS-LRR | sp|Q7XA39|RGA4_SOLBU | Putative disease resistance protein RGA4 |
| Vang0103s00400.1 | NBS-LRR | sp|O81825|DRL28_ARATH | Probable disease resistance protein At4g27220 |
| Vang0103s00320.1 | NBS-LRR | sp|Q9T048|DRL27_ARATH | Disease resistance protein At4g27190 |
| Vang0103s00300.1 | NBS-LRR | sp|Q9LRR4|R13L1_ARATH | Putative disease resistance RPP13-like protein 1 |
| Vang0103s00290.1 | NBS-LRR | sp|Q9LRR4|R13L1_ARATH | Putative disease resistance RPP13-like protein 1 |
| Vang0103s00270.1 | NBS-LRR | sp|Q9LRR4|R13L1_ARATH | Putative disease resistance RPP13-like protein 1 |
| Vang0103s00210.1 | NBS-LRR | sp|Q9LRR4|R13L1_ARATH | Putative disease resistance RPP13-like protein 1 |
| Vang0103s00030.1 | NBS-LRR | sp|Q9LRR4|R13L1_ARATH | Putative disease resistance RPP13-like protein 1 |
| Vang0071ss00490.1 | NBS-LRR | sp|Q40392|TMVRN_NICGU | TMV resistance protein N |
| Vang0071ss00440.1 | NBS-LRR | sp|O82500|Y4117_ARATH | Putative disease resistance protein At4g11170 |
| Vang0063ss00420.1 | NBS-LRR | sp|Q40392|TMVRN_NICGU | TMV resistance protein N |
| Vang0045ss00890.1 | NBS-LRR | sp|Q9T048|DRL27_ARATH | Disease resistance protein At4g27190 |
| Vang0045ss00880.1 | NBS-LRR | sp|Q9T048|DRL27_ARATH | Disease resistance protein At4g27190 |
| Vang0039ss01170.1 | NBS-LRR | sp|Q39214|RPM1_ARATH | Disease resistance protein RPM1 |
| Vang0039ss01030.1 | NBS-LRR | sp|Q40392|TMVRN_NICGU | TMV resistance protein N |
| Vang0033ss01460.1 | NBS-LRR | sp|Q40392|TMVRN_NICGU | TMV resistance protein N |
| Vang0033ss01420.1 | NBS-LRR | sp|Q40392|TMVRN_NICGU | TMV resistance protein N |
| Vang0029ss00280.1 | NBS-LRR | sp|O81825|DRL28_ARATH | Probable disease resistance protein At4g27220 |
| Vang0022ss04730.1 | NBS-LRR | sp|Q7XA40|RGA3_SOLBU | Putative disease resistance protein RGA3 |
| Vang0022ss04710.1 | NBS-LRR | sp|Q7XA40|RGA3_SOLBU | Putative disease resistance protein RGA3 |

| **Supplementary Table S21.** List of the loci used for phylogenetic tree construction by bayesian MCMC method | |
| --- | --- |
| Genename | Description |
| Vang0005s00360.1 | DUF538 domain containing protein, putative, expressed |
| Vang0010ss00510.1 | oxidoreductase, aldo/keto reductase family protein, putative, expressed |
| Vang0010ss01770.1 | OsAPx2 - Cytosolic Ascorbate Peroxidase encoding gene 4,5,6,8, expressed |
| Vang0022ss03920.1 | RNA-binding region RNP-1, putative, expressed |
| Vang0022ss04260.1 | ATP-grasp domain containing protein, expressed |
| Vang0026ss00190.1 | glyoxalase family protein, putative, expressed |
| Vang0029ss00480.1 | EF hand family protein, putative, expressed |
| Vang0033ss01320.1 | expressed protein |
| Vang0038s00460.1 | vignain precursor, putative, expressed |
| Vang0041s00170.1 | pentatricopeptide, putative, expressed |
| Vang0064ss00630.1 | lactate/malate dehydrogenase, putative, expressed |
| Vang0065s00200.1 | None |
| Vang0066ss00250.1 | ferredoxin--nitrite reductase, putative, expressed |
| Vang0069ss01010.1 | phospholipase D, putative, expressed |
| Vang0126s00150.1 | emp24/gp25L/p24 family protein, putative, expressed |
| Vang0134s00010.1 | glycosyl hydrolases family 16, putative, expressed |
| Vang0155s00030.1 | ras-related protein, putative, expressed |
| Vang0172s00440.1 | OsFBL5 - F-box domain and LRR containing protein, expressed |
| Vang01g03080.1 | microtubule associated protein, putative, expressed |
| Vang01g03640.1 | ras-related protein, putative, expressed |
| Vang01g05270.1 | glutathione S-transferase, putative, expressed |
| Vang01g06870.1 | peptide transporter PTR2, putative, expressed |
| Vang0295s00150.1 | major facilitator superfamily domain-containing protein 5, putative, expressed |
| Vang02g00230.1 | clathrin adaptor complex small chain domain containing protein, expressed |
| Vang02g02040.1 | peroxiredoxin, putative, expressed |
| Vang03g01140.1 | expressed protein |
| Vang03g03790.1 | heavy metal associated domain containing protein, expressed |
| Vang03g05690.1 | stem-specific protein TSJT1, putative, expressed |
| Vang03g05920.1 | ribosomal RNA large subunit methyltransferase J, putative, expressed |
| Vang03g14020.1 | Core histone H2A/H2B/H3/H4 domain containing protein, putative, expressed |
| Vang04g00250.1 | UDP-glucose 6-dehydrogenase, putative, expressed |
| Vang04g05520.1 | ELMO/CED-12 family protein, putative, expressed |
| Vang05g05120.1 | ctr copper transporter family protein, putative, expressed |
| Vang05g05620.1 | None |
| Vang05g06060.1 | miro, putative, expressed |
| Vang05g06100.1 | mitochondrial import inner membrane translocase subunit Tim17, putative, expressed |
| Vang05g07840.1 | tRNA methyltransferase, putative, expressed |
| Vang05g08700.1 | acyl-protein thioesterase, putative, expressed |
| Vang06g06160.1 | basic helix-loop-helix, putative, expressed |
| Vang06g07220.1 | tetraspanin family protein, putative, expressed |
| Vang06g07770.1 | expressed protein |
| Vang06g16180.1 | expressed protein |
| Vang06g18620.1 | expressed protein |
| Vang07g01190.1 | ADP-ribosylation factor, putative, expressed |
| Vang07g02780.1 | ADP-ribosylation factor, putative, expressed |
| Vang07g05830.1 | None |
| Vang07g06000.1 | S10/S20 domain containing ribosomal protein, putative, expressed |
| Vang07g06020.1 | AGAP003371-PA, putative, expressed |
| Vang07g06040.1 | anthranilate phosphoribosyltransferase, putative, expressed |
| Vang07g07160.1 | cytochrome c, putative, expressed |
| Vang08g01370.1 | IPR009009,IPR014733 |
| Vang09g03090.1 | cyclin, putative, expressed |
| Vang10g05040.1 | magnesium-protoporphyrin O-methyltransferase, putative, expressed |
| Vang10g05390.1 | tetraspanin family protein, putative, expressed |
| Vang10g06760.1 | aquaporin protein, putative, expressed |
| Vang10g06850.1 | genetic modifier, putative, expressed |
| Vang11g06740.1 | OsCML7 - Calmodulin-related calcium sensor protein, expressed |
| Vang11g09480.1 | nucleoside transporter, putative, expressed |
| Vang11g13370.1 | DEAD-box ATP-dependent RNA helicase, putative, expressed |
| Vang11g17350.1 | WD repeat-containing protein 5, putative, expressed |

| **Supplementary Table S23**. Raw NGS data for this study | |  |
| --- | --- | --- |
| **NCBI SRA ID** | **Description** | **Uploaded contents** |
| SRP049702 | Sequence of *Vigna angularis* var. *angularis* | Raw sequence reads of *V. angularis* var. *angularis* genome assembly |
| SRP049742 | Resequencing of wild adzuki bean | Raw sequence reads of *V. angularis* var. *nipponensis*, *V. nepalensis*, *V. nakashimae* |
| SRP049637 | *Vigna angularis* GBS | GBS data of *Vigna angularis* 133 lines |
|  |  |  |
